# In vitro and in vivo accumulation of magnetic nanoporous silica nanoparticles on implant materials with different magnetic properties

**DOI:** 10.1186/s12951-018-0422-6

**Published:** 2018-11-27

**Authors:** Hilke Catherina Janßen, Dawid Peter Warwas, David Dahlhaus, Jessica Meißner, Piriya Taptimthong, Manfred Kietzmann, Peter Behrens, Janin Reifenrath, Nina Angrisani

**Affiliations:** 1 NIFE-Lower Saxony Centre for Biomedical Engineering, Implant Research and Development, Clinic for Orthopedic Surgery, Hannover Medical School, Stadtfelddamm 34, 30625 Hannover, Germany; 2 Institute for Inorganic Chemistry, Leibniz University Hannover, Callinstraße 9, 30167 Hannover, Germany; 3 Institute of Pharmacology, Toxicology and Pharmacy, University of Veterinary Medicine, Foundation, Bünteweg 17, 30559 Hannover, Germany; 4 Institute of Micro Production Technology, Leibniz University Hannover, An der Universität 2, 30823 Garbsen, Germany

**Keywords:** Drug targeting, Core–shell nanoparticles, Superparamagnetic Fe_3_O_4_, Nanoporous silica, PEGylation, Biocompatibility, Ferritic steel, Martensitic steel, Mouse model

## Abstract

**Background:**

In orthopedic surgery, implant-associated infections are still a major problem. For the improvement of the selective therapy in the infection area, magnetic nanoparticles as drug carriers are promising when used in combination with magnetizable implants and an externally applied magnetic field. These implants principally increase the strength of the magnetic field resulting in an enhanced accumulation of the drug loaded particles in the target area and therewith a reduction of the needed amount and the risk of undesirable side effects. In the present study magnetic nanoporous silica core–shell nanoparticles, modified with fluorophores (fluorescein isothiocyanate/FITC or rhodamine B isothiocyanate/RITC) and poly(ethylene glycol) (PEG), were used in combination with metallic plates of different magnetic properties and with a magnetic field. In vitro and in vivo experiments were performed to investigate particle accumulation and retention and their biocompatibility.

**Results:**

Spherical magnetic silica core–shell nanoparticles with reproducible superparamagnetic behavior and high porosity were synthesized. Based on in vitro proliferation and viability tests the modification with organic fluorophores and PEG led to highly biocompatible fluorescent particles, and good dispersibility. In a circular tube system martensitic steel 1.4112 showed superior accumulation and retention of the magnetic particles in comparison to ferritic steel 1.4521 and a Ti90Al6V4 control. In vivo tests in a mouse model where the nanoparticles were injected subcutaneously showed the good biocompatibility of the magnetic silica nanoparticles and their accumulation on the surface of a metallic plate, which had been implanted before, and in the surrounding tissue.

**Conclusion:**

With their superparamagnetic properties and their high porosity, multifunctional magnetic nanoporous silica nanoparticles are ideal candidates as drug carriers. In combination with their good biocompatibility in vitro, they have ideal properties for an implant directed magnetic drug targeting. Missing adverse clinical and histological effects proved the good biocompatibility in vivo. Accumulation and retention of the nanoparticles could be influenced by the magnetic properties of the implanted plates; a remanent martensitic steel plate significantly improved both values in vitro. Therefore, the use of magnetizable implant materials in combination with the magnetic nanoparticles has promising potential for the selective treatment of implant-associated infections.

**Electronic supplementary material:**

The online version of this article (10.1186/s12951-018-0422-6) contains supplementary material, which is available to authorized users.

## Background

Depending on factors like location, time after surgery, or patient related risk factors, implant associated infections in arthroplasty/revision arthroplasty (up to 4/20%) and fracture repair (up to 7%; up to 40% for open fractures) represent a severe and prevailing complication in orthopedic surgery [1–4]. The implant surfaces provide conditions especially for biofilm-forming bacteria to adhere and colonize on these medical devices. Although those bacteria have been identified as a substantial cause for persistent infections since more than two decades [5, 6], the understanding of shielding mechanisms is still incomplete and the search for therapeutic strategies the center of many studies. Beside prophylactic approaches like, e.g., routine antibiotic treatment or the development of antimicrobially enhanced implant surfaces [7], specific therapeutic approaches are in demand. Especially in the light of proceeding chemoresistance, the focus lies on reduced antibiotic drug levels applied as effectively as possible.

Apart from directly equipping the implant surface with antimicrobial delivery systems [8, 9], possible solutions lie in the use of nanoparticles which either deliver antibiotics only when an infection is present [10], or which are specifically targeted to the infection site and are applied only if necessary. Targeting can in principle be achieved in similar ways as has been described for tumor diagnosis and treatment in the last years. However, doubts have arisen whether the classical targeting strategies really work [11]. An alternative are magnetic nanoparticles which can be guided to the infection site by an externally applied magnetic field [12–14]. This idea, however, becomes problematic when the infection site or implant is buried deep within the body. In the present concept, we use magnetizable implants which are able to increase the strength of the externally applied magnetic field in an area localized around the implant. Consequently, efficient targeting of magnetic nanoparticles to the implant should be possible. With this concept of implant-directed magnetic drug targeting (ID-MDT), lower amounts of particles and thus drugs would be necessary to reach sufficient concentrations in the infection area. Accordingly, the risk of undesirable side effects could be reduced and treatment of the infection could be improved. We have already shown the general working principle in an ex vivo laboratory set-up [14]. Altogether, this novel concept constitutes a promising, future strategy for the treatment of implant-associated infections by drug-loaded magnetic nanoparticles in combination with a magnetizable implant.

For in vivo application, the magnetic nanoparticles to be employed should combine good biocompatibility and large cargo space. Core–shell nanoparticles with a magnetic core and a nanoporous shell are able to store large amounts of drug and represent a promising group of materials [15, 16]. The outer surface of the shell can further be used to regulate the dispersibility, colloidal stability and other features of the nanoparticles. Especially magnetic nanoporous silica nanoparticles (MNPSNPs) are promising candidates as drug carriers for a selective treatment of infections. In such a particle, a superparamagnetic magnetite NP core is combined with a nanoporous silica shell with large porosity for drug delivery and very good biocompatibility, thus excellent prerequisites for utilization in biomedical applications. The possibility for further functionalization of the nanoporous silica shell via post-grafting with silanes with a diversity of functional groups further increases the multifunctionality. Regarding this, the coupling of organic fluorescent dyes for bioimaging and the so-called PEGylation with a PEG-silane (Poly(ethylene glycol)-silane) for an improved biocompatibility and biodistribution behavior are part of the toolbox for installing multifunctionality on MNPSNPs [17].

In the present study we first examine whether plates with different magnetic properties exhibit different levels of nanoparticle accumulation capability in vitro. Then, the biocompatibility of the (functionalized) MNPSNPs is tested in vitro on cell lines NIH-3T3 and HepG2 with regard to viability and proliferation. Most importantly, we study the in vivo distribution and biocompatibility of the MNPSNPs as well as their accumulation at a ferritic plate compared to a paramagnetic control after subcutaneous injection in a mouse model.

## Methods

### Synthesis of magnetic nanoporous silica nanoparticles (MNPSNPs)

For the synthesis of MNPSNPs, hydrophobic magnetite nanoparticles are produced first. In a second step, these particles were enveloped with a nanoporous silica shell, which in principal enables the storage of drugs, but is used here for the attachment of fluorescent dyes, namely fluorescein isothiocyanate (FITC) or rhodamine B isothiocyanate (RITC), and the modification with a PEG-silane.

For the syntheses all chemicals were used without further purification. Iron(II) chloride tetrahydrate (≥ 99%), iron(III) chloride hexahydrate (99%), oleic acid (90%), chloroform (≥ 99%), cetyltrimethylammonium bromide (CTAB, ≥ 98%), Ammonium hydroxide solution (≥ 25% NH_3_ in H_2_O) tetraethyl orthosilicate (TEOS, ≥ 99%), ethyl acetate (99.8%), 3-aminopropyl trimethoxysilane (APTMS, 97%), fluorescein isothiocyanate isomer I (FITC, ≥ 90%), rhodamine B isothiocyanate mixed isomers (RITC) were purchased from Sigma-Aldrich Corporation (München, Germany). Absolute ethanol (≥ 99.5%) was purchased from Merck (Darmstadt, Germany). [Hydroxy(polyethyleneoxy)propyl] triethoxysilane (PEG-Silane, MW 575–750 g mol^−1^, 50% in ethanol) was purchased from Gelest (Morrisville PA, USA). Ultrapure water (18.2 MΩ cm) was used in all chemical operations.

#### Synthesis of hydrophobic magnetite (Fe_3_O_4_) NPs

Hydrophobic magnetite (Fe_3_O_4_) NPs were synthesized with a co-precipitation method based on a published procedure with some modifications [17]. In a first step, 30 mL of ultrapure water was purged with nitrogen gas for 10 min. Afterwards 4.80 g (18 mmol) FeCl_3_·6H_2_O, 2.00 g (10 mmol) FeCl_2_·4H_2_O and 0.8 mL (3 mmol) oleic acid were added to the water under vigorous stirring and nitrogen atmosphere. The reaction mixture was heated to 90 °C under stirring. After the rapid addition of 20 mL ammonium hydroxide (14 wt%, 302 mmol) the mixture turned black immediately. The reaction mixture was stirred for 2.5 h at 90 °C and afterwards cooled down to room temperature. The black precipitate was magnetically separated and washed three times with ultrapure water before being dried in a vacuum oven at room temperature.

#### Synthesis of magnetic nanoporous silica nanoparticles (MNPSNPs)

MNPSNPs were synthesized according to a published procedure with some modifications [18]. 22.5 mg of hydrophobic oleic acid-capped Fe_3_O_4_ NPs were dispersed in 3 mL (37 mmol) of chloroform in an ultrasonic bath. This mixture was added under vigorous stirring to 30 mL of an aqueous solution containing 0.3 g (1 mmol) CTAB. To carry out a phase transfer from the organic to the aqueous phase, the chloroform was evaporated from the CTAB-Fe_3_O_4_ dispersion for 1 h at 65 °C. After the addition of 270 mL of ultrapure water, the mixture was stirred for 0.5 h at 40 °C. Then, 9 mL (231 mmol) of ammonium hydroxide (≥ 25 wt%), 1.5 mL (7 mmol) of TEOS and 15 mL (154 mmol) of ethyl acetate were added within 1 min under vigorous stirring. The reaction mixture was stirred for 3 h at 40 °C. After cooling to room temperature, the light brown product was obtained by centrifugation. The particles were washed three times with absolute ethanol before being dried under vacuum. For the removal of the remaining surfactant, the MNPSNPs were calcined, using a heating rate of 1 °C min^−1^ to reach 550 °C where the sample was kept for 5 h. These particles are denoted as MNPSNPs.

#### Modification of MNPSNPs with PEG and FITC or RITC

The modification of MNPSNPs with PEG and FITC or RITC is based on a published procedure with some modifications [17]. APTMS was coupled in a co-condensation reaction with FITC to FITC-APTMS or with RITC to RITC-APTMS [19]. These thioureas were prepared by combining 18 mg (0.05 mmol) FITC or 25 mg (0.05 mmol) RITC, 2 mL absolute ethanol and 5 µL APTMS under continuous stirring and exclusion of light for 24 h. In a further step, the MNPSNPs were modified via the post-grafting method [18]. For this purpose, 120 mg of MNPSNPs were dispersed in 42 mL of absolute ethanol in an ultrasonic bath. Then the suspension was heated to 50 °C under vigorous stirring and 502 µL of FITC-APTMS or RITC-APTMS and 1.08 mL of PEG-silane was added to the particles. The mixture was stirred with exclusion of light for 24 h at 50 °C. After cooling to room temperature, the modified particles were obtained by centrifugation. They were furthermore washed four times with absolute ethanol before being dried under vacuum and dark conditions. FITC- and PEG-modified particles are denoted as MNPSNP@FITC-PEG; RITC and PEG modified particles are denoted as MNPSNP@RITC-PEG.

### Characterization of Fe_3_O_4_ NPs and MNPSNPs

Transmission electron microscopy (TEM) was performed with a FEI Tecnai G2 F20 TMP instrument (*C*_S_ = 2 mm, *C*_C_ = 2 mm) with a 200 kV field emission gun in bright-field mode. For sample preparation, 400-mesh carbon-coated copper grids (Quantifoil) were used. The samples were dispersed via ultrasonication, dropped on the grid and dried. Particle size distributions were evaluated with NIH ImageJ. Fourier-transform infrared spectroscopy (FT-IR) was measured with a Bruker Tensor 27 instrument in transmission after preparation of KBr pellets (Sigma Aldrich). N_2_ physisorption measurements were performed on a Quantachrome Autosorb-3 instrument after outgassing of the sample in vacuum for 24 h at 100 °C. Surface areas and pore sizes were calculated with the software ASiQwin (version 2.0) from Quantachrome, applying the Brunauer–Emmett–Teller (BET) equation and the density functional theory (DFT), respectively. The experimental data were fitted to the Quantachrome Kernel “N_2_ at 77 K on silica (cylinder/sphere pore, NLDFT ads. Model). For the determination of the total pore volumes the single point method at *p*/*p*_0_ = 0.95 was used. Thermogravimetric analysis (TGA) was measured with a Netzsch STA 409 PC/PG thermoanalyzer. The samples were heated with a rate of 5 °C/min in an Al_2_O_3_ crucible. As flushing gas a mixture of Ar and O_2_ (80% Ar, 20% O_2_) was used and the measurements were terminated at 1000 °C. Room temperature magnetization curves were measured using a Lake Shore, Inc., Model 7407 vibrating sample magnetometer (VSM). Sample holders made of PLEXIGLAS GS/XT were created. They have geometries similar to 730931 Kel-F bulk/powder upper/bottom cup and their compartment volume for holding powder sample is 30 mm^3^. The average weight of samples was 6 mg. Two measurement ranges, i.e. a high field range (15 kG) and a low field range (100 G), were employed to measure saturation and residual magnetization, the so-called remanence, of the samples, respectively. The complete hysteresis loop measurements were performed starting from the positive maximum field to the negative maximum field and back to the positive maximum field. The ramp rates of the field are 75 G s^−1^ and 5 G s^−1^ for the high and the low field ranges, respectively. Hydrodynamic diameter and zeta potential were measured by dynamic light scattering (DLS) with a Zetasizer Nano ZS. The samples were dispersed in ultrapure water in an ultrasonic bath resulting in suspensions with a mass concentration of 230 µg mL^−1^.

### Cell culture

The cell lines NIH-3T3 and HepG2 (both CLS, Germany) were maintained under standard cell culture conditions in a humidified 5% CO_2_ atmosphere at 37 °C in 25 cm^2^ flasks (Greiner, Germany).

The murine fibroblast cell line NIH-3T3 was cultured with DMEM/Ham’s F12 medium, supplemented with 10% FCS and Penicillin (100 units/mL)/Streptomycin (100 µg/mL) (all Biochrom, Germany). The humane hepatoma cell line HepG2 was cultured with Ham’s F12 medium (Biochrom, Germany), supplemented with 10% FCS, Penicillin (100 units/mL)/streptomycin (100 µg/mL) and 2 mM l-glutamine (Life Technologies, USA).

For cell proliferation testing, 10.000 NIH-3T3 and 35.000 HepG2 cells per well, respectively, were seeded in 96-well microtiter plates. On the following day, these were treated with MNPSNPs. For cell viability testing, cells were grown to full confluency for 2–5 days before the treatment. Incubation times with the MNPSNPs were 24 and 48 h.

The MNPSNPs were suspended in aqua bidestillata and then mixed 1:10 with cell culture medium to obtain the final concentrations of 0 (control group), 5, 10, 25, 50 and 100 µg MNPSNPs per mL complete cell culture medium. The negative controls demonstrate an intentional kill of cells and therefore contain cell culture medium with 5% dimethylsulfoxide (DMSO) (Merck, Germany).

#### Cell proliferation

For measuring the cell proliferation a crystal violet staining assay was performed. After incubation the supernatant was removed and the cells were fixed with 2% glutaraldehyde (Sigma, Germany) in PBS (NaCl 8 g/L, KCl 0.2 g/L, Na_2_HPO_4_ × 2H_2_O 1.44 g/L, KH_2_PO_4_ 0.2 g/L, in aqua bidestillata; pH 7.4) for 20 min and then stained with 0.1% crystal violet (Merck, Germany) in aqua bidestillata for 30 min. After a washing and a drying step, the dye was dissolved in 100 µL of 2% Triton ×-100 (Sigma, Germany) in aqua bidestillata per well. Finally, the absorbance at 570 nm was measured with a MRX microplate-reader.

#### Cell viability

After incubation the supernatant was removed and a 1:6 solution of CellTiter 96^®^ MTS (Promega, Germany) and cell culture medium was added. After 1 h the absorbance at 490 nm was measured with a MRX microplate-reader (Dynatech, Germany).

### Accumulation of MNPSNPs at different implant materials in vitro

#### Implant materials

Three different implant materials were used to produce 6 × 2 × 1 mm plates: stainless steel 1.4122 as a martensitic material (M), stainless steel 1.4521 as a ferritic material (F) and the titanium alloy Ti90Al6V4 (T) as paramagnetic control. Both stainless steels are ferromagnetic but with different properties. Martensitic steel provides high remanence while the relative permeability is low. On the contrary, ferritic steel combines high relative permeability with very low remanence. The paramagnetic titanium alloy Ti90Al6V4 is an established implant material in orthopedics with proven biocompatibility and serves as control.

The ferritic steel was provided by outokumpu Nirosta GmbH (Germany) as 1 mm thin sheet. The titanium alloy was supplied by GoodFellow (England) as 50 × 50 × 1 mm sheet. The martensitic steel plates were produced from a cylinder (Sürth Stahl- und Metallhandel, Germany). First, a 1 mm thin roundly shaped plate was cut by wire-electro discharge machining (AD325L, Sodick Deutschland GmbH, Germany) wire ø 0.25 mm, cutting speed 2 mm min^−1^). The temperature-neutral machining technique does not influence the magnetic properties of the material. The plates were cut by aqua jet cutting (Microwaterjet F4, MDC Max Daetwyler AG, Switzerland, nozzle ø 0.12 mm, water pressure 330 MPa, feed rate 63 and 78 mm min^−1^ for steel and titanium alloy, respectively) at the Unterwassertechnikum (Institute of Materials Science, Leibniz University Hannover) with a final size of 6 × 2 × 1 mm. Implants were grinded with a sand paper to erase sharp edges, then cleaned using acetone and ultrasound.

#### In vitro setup

**Fig. 1:**
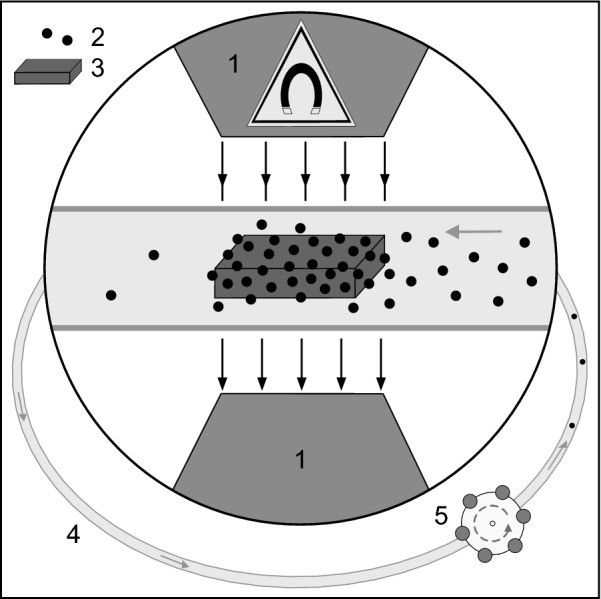
In vitro setup: (1) electromagnet, (2) FITC-linked MNPSNPs, (3) plate, (4) Heidelberger extension, (5) peristaltic pump

To examine the influence of the different plate materials a closed circuit tube system was set up (Fig. 1). A Heidelberger extension (140 cm, inner diameter 3 mm, Fresenius Kabi AG, Germany) was connected to a 2-stop tubing for peristaltic pumps (Tygon Standard R-3607, violet–violet, inner diameter 2.06 mm, Ismatec/Cole Parmer GmbH, Germany) via two Luer-Lock connections and a three-way stopcock (Discofix^®^-3, B.Braun Melsungen AG, Germany).

FITC-linked MNPSNPs were dispersed in distilled water (A. dest.) to prepare a stock suspension of 3000 µg mL^−1^. 1 mL of this suspension was added to 12 mL A. dest. to get the loading suspension with a mass concentration of 230 µg mL^−1^. This concentration corresponded to the nanoparticle mass used for in vivo examinations. For each cycle, one plate was placed into the Heidelberger extension and the whole tube system was filled with the loading suspension via the three-way stopcock, carefully avoiding the creation of air bubbles. In total, n = 5 ferritic and titanium alloy plates, respectively, were tested as well as n = 7 martensitic plates. The part of the Heidelberger extension which contains the plate was placed between the poles of an electro magnet (EM2, Magnet-Messtechnik J. Ballanyi, Germany). To evaluate the accumulation capability of the magnetic field alone, 7 cycles were performed without any plate in the tube system. The distance between the pole shoes was 13 mm, the magnetic field strength near the plates/tube 1.8 T. The MNPSNP suspension was circulated by a peristaltic tube pump (IPC-N-8, Ismatec/Cole Parmer GmbH, Germany) with a flow rate of 5.2 mL min^−1^ resulting in a flow velocity of 12.2 mm s^−1^ within the Heidelberger extension. The circulation was maintained for 10 min. Afterwards, the 40 mm long part of the Heidelberger extension which was situated between the pole shoes was isolated with two clamps. The containing MNPSNP suspension and plate—if present—were transferred to an Eppendorf tube^®^ (0.5 mL, Eppendorf AG, Germany, sample a) and treated by ultrasound in an ultrasonic bath (Elma Schmidbauer GmbH, Germany) for 5 min. Subsequently, the plate was carefully transferred into another Eppendorf tube^®^ with 150 µL A. dest. (sample b). The first Eppendorf tube^®^ containing MNPSNPs which were accumulated in the suspension plus MNPSNPs that were detached from the plate by ultrasound was again placed in the ultrasound bath for 2 min and the suspension (sample a) then analyzed using a multi-detection microplate reader (Synergy™2, BioTek Instruments, GmbH, Germany). The second Eppendorf tube^®^ (sample b) with suspension and plate was treated by ultrasound for 5 min. The suspension was extracted using a pipet and analyzed with the Synergy™ 2. The Eppendorf tube^®^ with enclosed plate was refilled with 150 µL A. dest. and again treated by ultrasound for 5 min. The procedure was repeated until the resulting values were below the detection limit. For cycles without plate in the tube system, results are based solely on sample a.

To evaluate the remanence of the plates, the experiment was repeated for martensitic (n = 7), ferritic (n = 6) and titanium alloy plates (n = 5) with an additional circulation time of 3 min after the electromagnet was turned off.

#### Calculation of accumulated MNPSNP mass

For the calculation of the accumulated MNPSNP mass, the fluorescence intensity of the following suspensions was taken into account: stock suspension, serial dilutions from stock suspension, loading suspension, suspension of Eppendorf tube^®^ 1(a) and suspension of any following Eppendorf tube^®^ until the detection limit of 8000 was reached.

100 µL each were transferred to a 96-microwell plate and placed in the microplate reader. Before the measurement, the microwell plate was shaken fastly for 5 s. Excitation was performed at 485 nm. Intensity of the emission at 528 nm and room temperature (RT) was measured.

As computation base, a 1:1.5 serial dilution of the stocking base was performed and the resulting fluorescence intensity graphically plotted (x-axis; y-axis: mass concentration) using Excel (version 14.0, Microsoft Office Profession Plus 2010). Thereof, a fourth degree polynomial function was calculated with a coefficient of determination of R^2^ = 0.9998 and R^2^ = 1, respectively.

All obtained fluorescence values were processed using the polynomic function to calculate the mass concentration of MNPSNPs. Taken into account the respective volumes, the MNPSNP mass of each sample was calculated via the formula

$${\text{mass m }}\left[ {{\rm{\upmu g}}} \right]\, = \,{\text{mass concentration c }}\left[ {{\rm{\upmu g}} {\text{mL}}^{ - 1} \left] { \, *{\text{ volume V }}} \right[{\text{mL}}} \right].$$

To assess the influence of the different implant materials the percentage of total accumulated MNPSNP mass relating to MNPSNP mass in the loading suspension was calculated according to the following formula:

$${{\left( {{\text{m}}_{{\left( {\text{a}} \right) }} + {\text{m}}_{{\left( {\text{b}} \right) }} + {\text{m}}_{{({\text{y}})}} } \right)} \mathord{\left/ {\vphantom {{\left( {{\text{m}}_{{\left( {\text{a}} \right) }} + {\text{m}}_{{\left( {\text{b}} \right) }} + {\text{m}}_{{({\text{y}})}} } \right)} {{\text{m}}_{{({\text{loading suspension}})}} }}} \right. \kern-0pt} {{\text{m}}_{{({\text{loading suspension}})}} }}\,*\, 100$$

### In vivo examinations

The animal experiments were authorized according to the German Animal Welfare Act and registered as number 33.12-42502-04-13/1103. In total, five female BALB/cJHanZtm mice with an average body weight of 27.2 g were part of the experiments. Each mouse received subcutaneously one ferritic plate at the left hind leg and one titanium alloy plate as control at the right hind leg. Mouse husbandry was performed in small groups of up to 3 mice with 14 h/10 h-day/night cycle and non-restricted food and water access. The experiments were implemented as follows.

For peri-operative analgesia, meloxicam (1 mg/kg body weight (BW), s.c.) was administered. Anaesthesia was established by intraperitoneal injection of 0.1 mL of ketamine-xylazine-sodium chloride-mixture (ratio 0.8 mL NaCl 0.9% (B. Braun Melsungen AG, Germany), 0.2 mL ketamine 10% (Wirtschaftsgenossenschaft deutscher Tierärzte eG, Germany) and 0.1 mL xylazine 2% (CP-Pharma Handelsgesellschaft mbH, Germany)) resulting in a dose of 70 mg ketamine/kg BW and 7 mg xylazine/kg BW. To avoid eye desiccation, eye ointment was applied (Bepanthen^®^ Augen- und Nasensalbe, Bayer Vital GmbH, Germany). Both hind legs were shaved and Veet Hair Removal Cream (Reckitt Benckiser Deutschland GmbH) was used to achieve a hairless field of surgery. Afterwards, the skin was washed and disinfected (Kodan^®^ Schülke & Mayr GmbH, Germany).

If anaesthesia prolongation was necessary, midazolam (5 mg/kg BW) was administered intraperitoneally and eventually antagonized by flumazenil (0.5 mg/kg BW). The surgical procedure was the same for both hind legs: a 3 mm long incision was placed beginning 1–2 mm distal to the femoral head. The skin was detached from the underlying fasciae and muscles so that the plate could be situated distally of the incision parallel to the femur. Wound closure was performed with PROLENE^®^ 6-0 (Johnson & Johnson Medical GmbH Ethicon Germany). Immediately after implantation of both plates, 0.05 mL RITC-linked MNPSNPs with a concentration of 4600 µg mL^−1^ were injected subcutaneously onto each implant which resulted in 230 µg MNPSNPs/implant site.

Afterwards, the mouse was placed between the poles of the electromagnet for 10 min. Corresponding to the in vitro trial the distance between the pole shoes was approx. 13 mm and the magnetic field strength near the implanted plates 1.8 T. The mouse was placed under red light until recovering from anesthesia.

The post-surgical examination period was 1 week. The mice were examined clinically on a daily basis for food and water intake, alteration of weight and any plate associated changes. After this time, the animals were sacrificed by cervical dislocation. The plates were carefully removed. Immediately, blood samples were taken out of the medial corner of the eye using a capillary. Organ samples were collected and processed according to the regarding method.

#### MNPSNPs detection in tissues by fluorescence analysis and pathological changes of organs

Blood samples were dispensed onto slides for fluorescence analysis.

One piece (0.4 × 0.3 × 0.3 mm^3^) of liver, spleen, kidneys and lung of each animal as well as exemplary brain samples were chopped and smeared onto slides using a scalpel.

The remaining organs of these samples and samples of heart, lymph nodes (*Lnn. Iliaci*) and muscle and skin of the hind leg were fixed in 0.4% buffered formalin for 48–72 h and embedded in paraffin. 5 µm thin sections were cut with a rotary microtome (Leica RM 2155, Leica Biosystems, Germany).

All fluorescence analyses were performed using a Axioskop 40 with AxioCam MRc digital camera and Zeiss AxioVision software (Carl Zeiss AG, Germany) with 400-fold magnification using a red filter (filter set 20, Excitation BP 546/12, Beam Splitter FT 560, Emission BP 575-640, Carl Zeiss AG, Germany). A minimum of ten fields of view per slide were included in the evaluation depending on the homogeneity of MNPSNP distribution in the sample. Each field of view was scored regarding the MNPSNP quantity (none, occasional, few, many, plentiful) and cluster size (very small, small, medium, large, very large clusters, Table 1). Values were calculated per ten fields of views and added to a total score leading to a possible score range from 0 to 350.

**Table 1:** Score for fluorescence analysis of MNPSNP quantity in organ and blood samples as well as drop samples taken of plates (suspension 1–3)

Cluster size	Quantity				
	None (0)	Occasional (1–5)	Few (≤ 20)	Many (≤ 100)	Plentiful (> 100)
Very small	0	1	2	3	5
Small	0	2	3	4	6
Medium	0	3	4	5	7
Large	0	4	5	6	8
Very large	0	5	6	7	9

Histological slices of organ samples of liver, spleen, both kidneys, lungs, brain, lymph nodes (*Lnn. Iliaci*), and muscle and skin of both hind legs were analyzed both unstained and after standard hematoxylin–eosin (H.E.) staining according to established protocols. For fluorescence analysis the same settings were used as for blood and non-embedded samples. The distribution of MNPSNPs was assessed descriptively. H.E. stained slices were analyzed also descriptively for pathological changes of organ structure.

#### MNPSNP detection on explanted plates by fluorescence analysis

After explantation of plates with partially adhering tissue each plate was placed in an Eppendorf tube^®^ and 100 µL A. dest. were added. Accumulated MNPSNPs were detached by vortexing (1 min, 2000 cycles s^−1^, IKA^®^ MS 3 basic, IKA Works, Inc., USA) followed by ultrasonic treatment (5 min, TRANSSONIC 310 Elma^®^, Elma Schmidbauer GmbH, Germany). After transferring the plate into a second Eppendorf tube^®^ containing 100 µL A. dest. suspension of tube 1 was again treated by ultrasound for 2 min and then analyzed. The second Eppendorf tube^®^ was placed in the ultrasonic bath and treated for 5 min. The suspension was withdrawn and analyzed; the tube with the remaining plate was refilled with 100 µL A. dest. and the treatment was repeated. Five drops of each suspension were pipetted on a slide, dried at room temperature and scored regarding quantity and size of MNPSNP cluster according to the score used for organ smear samples (Table 1). The summed score regarding quantity and size of MNPSNP cluster (Table 1) was composed of 5 values for peripheral regions of the drops and 15 values from corresponding fields of view in the middle region of the drops for each suspension (1–3). The possible score range was 0–2100.

To evaluate eventually remaining MNPSNPs on the plates, the plates themselves were analyzed descriptively by fluorescence microscopic evaluation using the same settings as for the other samples. Distribution (margin/sites of the plates) and quantity (none, few, many) were assessed.

### Statistics

For cell proliferation and cell viability the experiments were performed 6 times with triplicate measurements. Analysis was performed with graphpad prism 6 (GraphPad Software, Inc., USA) using One-way ANOVA and Dunnett’s multiple comparisons test. *p < 0.05 was considered significant.

For fluorescence analysis of in vitro and in vivo experiments, statistics were performed using SPSS^®^ 25 (IBM, USA). After testing for normal distribution One-way ANOVA, student’s t-test and Kruskal–Wallis/Mann–Whitney-U tests were performed, respectively, with differences considered statistically significant if p < 0.05.

## Results

### Characterization of Fe_3_O_4_ NPs and MNPSNPs

**Fig. 2:**
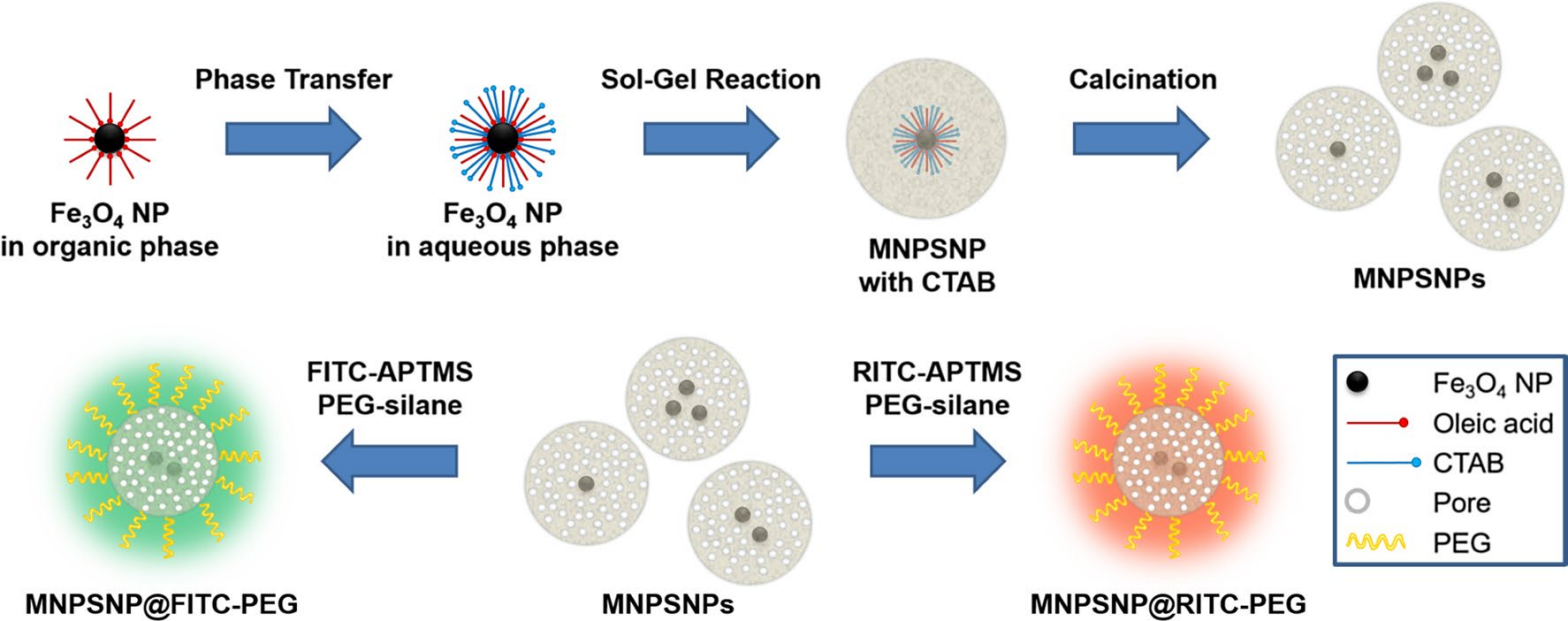
Top: Scheme of synthesis route for MNPSNPs, bottom: scheme for modification of MNPSNPs with PEG and FITC (left) or RITC (right)

The synthesis route for MNPSNPs based on hydrophobic oleic acid-capped Fe_3_O_4_ NPs and the following modification of MNPSNPs with PEG and FITC or RITC are illustrated in Fig. 2.

**Fig. 3:**
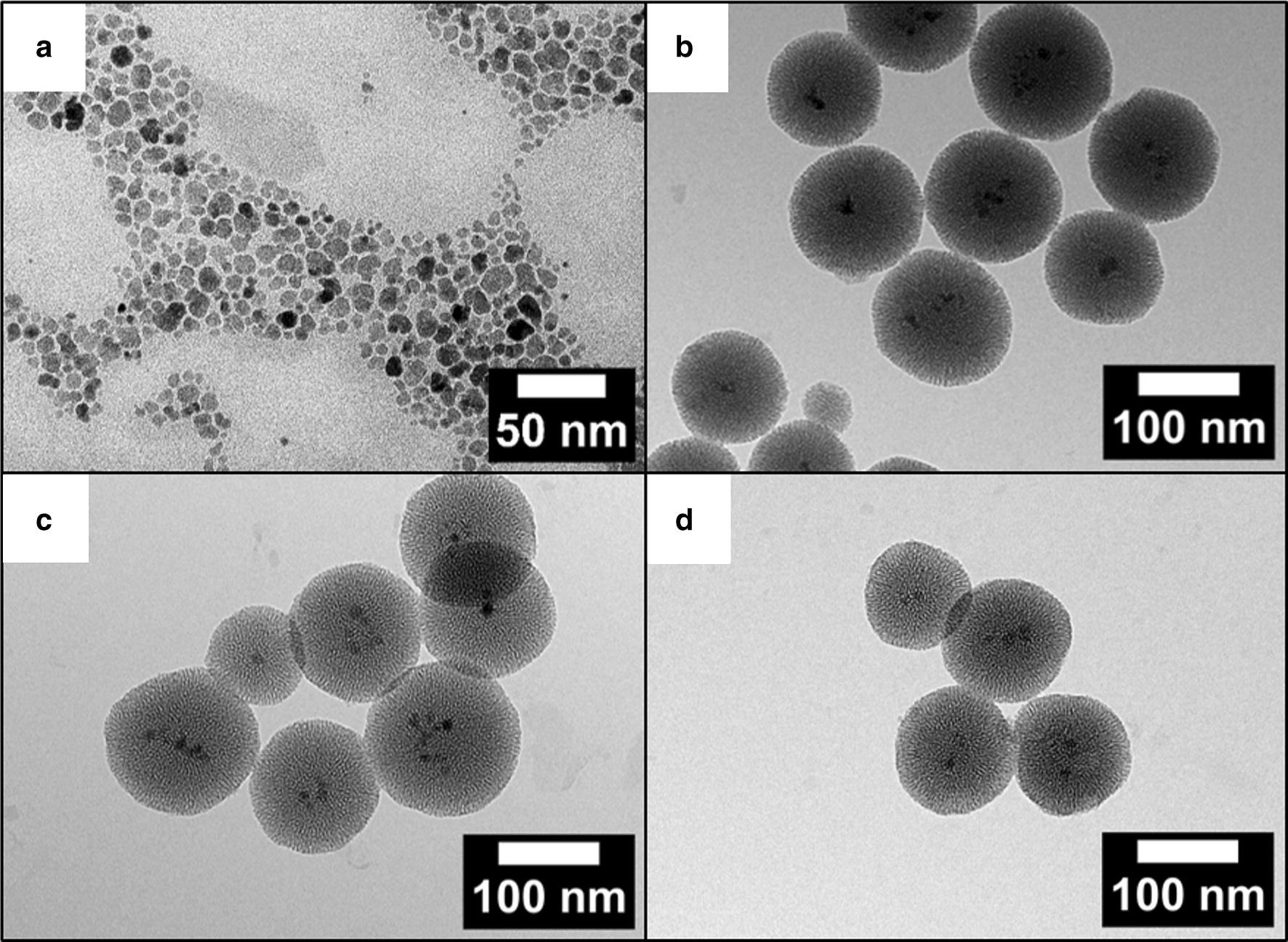
TEM images of (**a**) oleic acid-capped Fe_3_O_4_ NPs, (**b**) unmodified MNPSNPs, (**c**) MNPSNP@FITC-PEG, (**d**) MNPSNP@RITC-PEG

Based on TEM investigations the hydrophobic Fe_3_O_4_ NPs had an average particle diameter of 10 ± 2 nm (Fig. 3a). The particle size distribution is shown as a histogram in Figure S1 of Additional file 1.

**Fig. 4:**
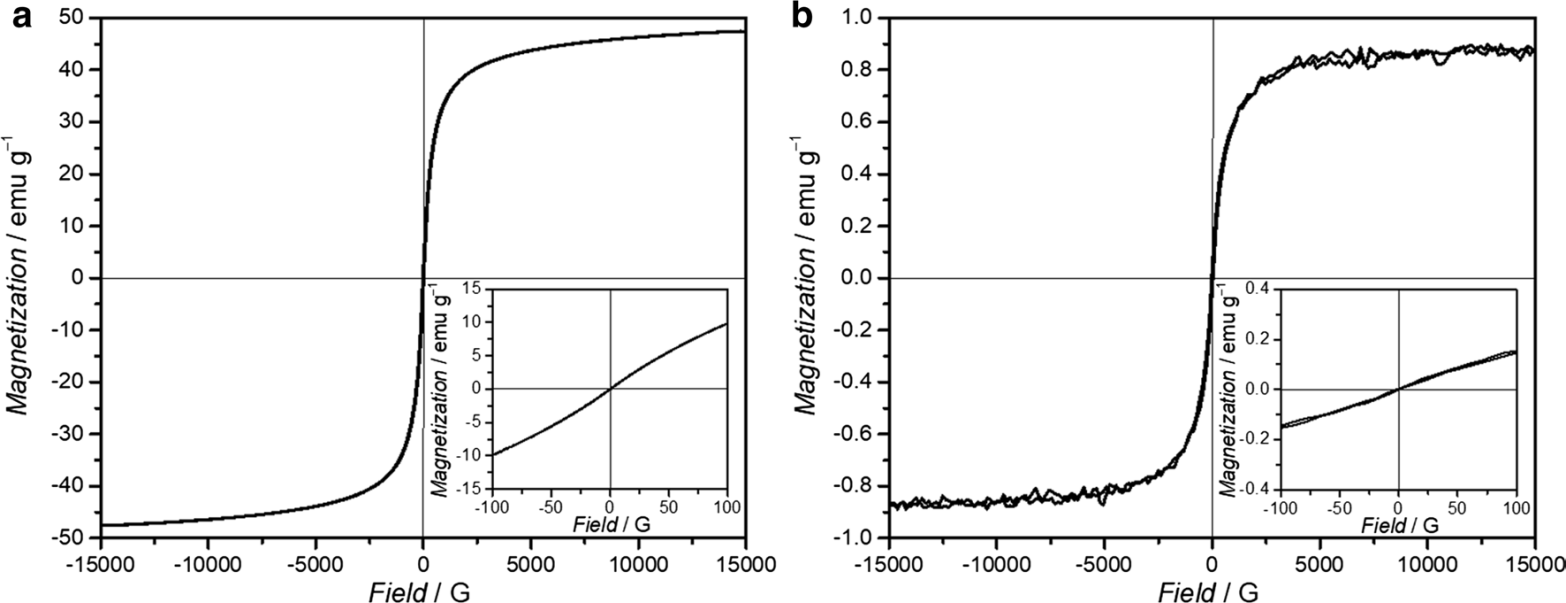
Magnetization curves at 300 K of (**a**) oleic acid-capped Fe_3_O_4_ NPs and (**b**) unmodified MNPSNPs. The inset in bottom right is the low-field magnetization curve

The Fe_3_O_4_ NPs as core material were stabilized with a total amount of 25% of oleic acid on the particle surface calculated from TGA results (Figure S2 of Additional file 1) Fig. 4a shows measurements of the room temperature magnetization of the hydrophobic Fe_3_O_4_ NPs with a vibrating-sample magnetometer (VSM). This curve presents a saturation magnetization of 48 emu g^−1^ at room temperature with characteristic superparamagnetic behavior with no remanence. Even when the applied magnetic field was small, no remanence was detected.

After phase transfer of CTAB-coated Fe_3_O_4_ NPs to the aqueous phase, the particles were used as seeds for the formation of a nanoporous silica matrix to create the MNPSNPs. In Fig. 3b uniform core–shell particles with a spherical shape and an average particle diameter of 112 ± 16 nm are presented. Inside the MNPSNPs multiple dark spots are visible indicating Fe_3_O_4_ NPs as cores. The room temperature magnetization curve of unmodified MNPSNPs is presented in Fig. 4b. Similar to the Fe_3_O_4_ core material, MNPSNPs showed no remanence in their magnetization curves and hence superparamagnetic behavior with a saturation magnetization of 1 emu g^−1^. The clear decrease of the saturation magnetization of the Fe_3_O_4_ core material from 48 to 1 emu g^−1^ is based on the smaller amount of magnetic material in the MNPSNPs with a silica shell thickness of 50 nm in comparison to the pure oleic-acid stabilized Fe_3_O_4_ NPs.

**Fig. 5:**
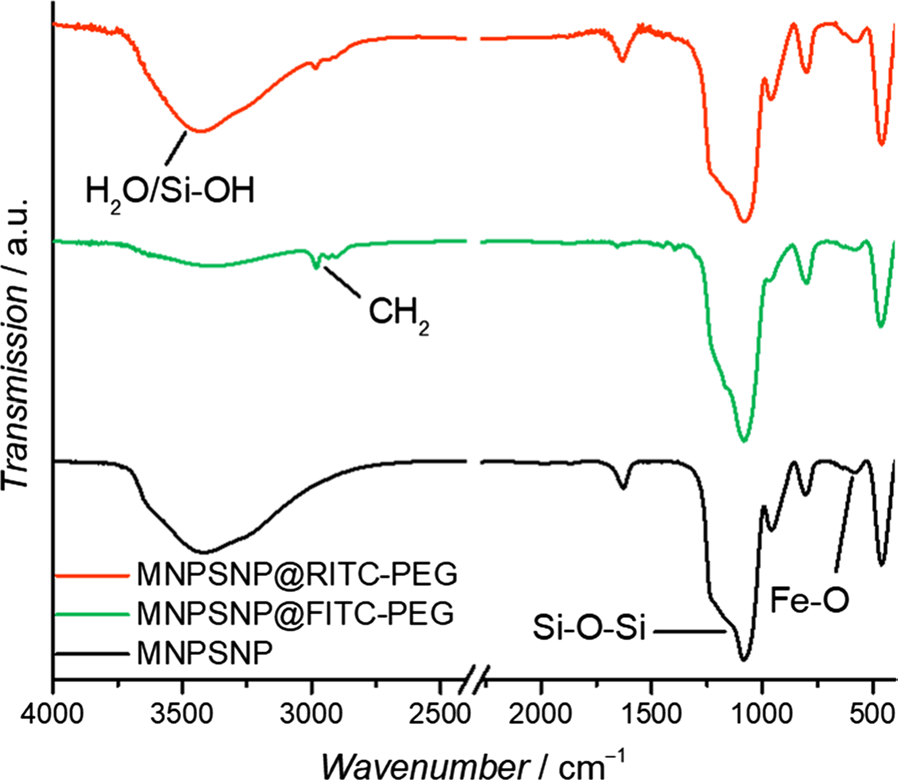
FT-IR spectra of unmodified MNPSNPs (black), MNPSNP@FITC-PEG (green) and MNPSNP@RITC-PEG (orange)

After modification with dye and PEG, the particles showed similar diameters and no loss of spherical shape so the characteristic composite structure of MNPSNPs was preserved (Fig. 3c, d). The particle size distributions of unmodified and modified MNPSNPs are presented as histograms in Figure S1 of Additional file 1. For investigations of the colloidal stability, the hydrodynamic diameter and size distribution with DLS as well as zeta potentials were measured in ultrapure water (Figure S3 and Table S1 of Additional file 1). In comparison to the TEM investigations, MNPSNPs showed larger particle diameters with a narrow size distribution for unmodified MNPSNPs (191 nm), MNPSNP@FITC-PEG (243 nm) and MNPSNP@RITC-PEG (220 nm) due to the hydration layer in aqueous medium. The increase of the particle diameter for modified MNPSNPs is based on the modification with the dye and outer PEG layer. In addition, zeta potential measurements revealed strong negative surface charges in the range of − 30 mV, which indicates colloidal stability and thus the prevention of aggregation due to electrostatic repulsion. FT-IR spectra of unmodified and modified MNPSNPs with dye and PEG are depicted in Fig. 5.

**Fig. 6:**
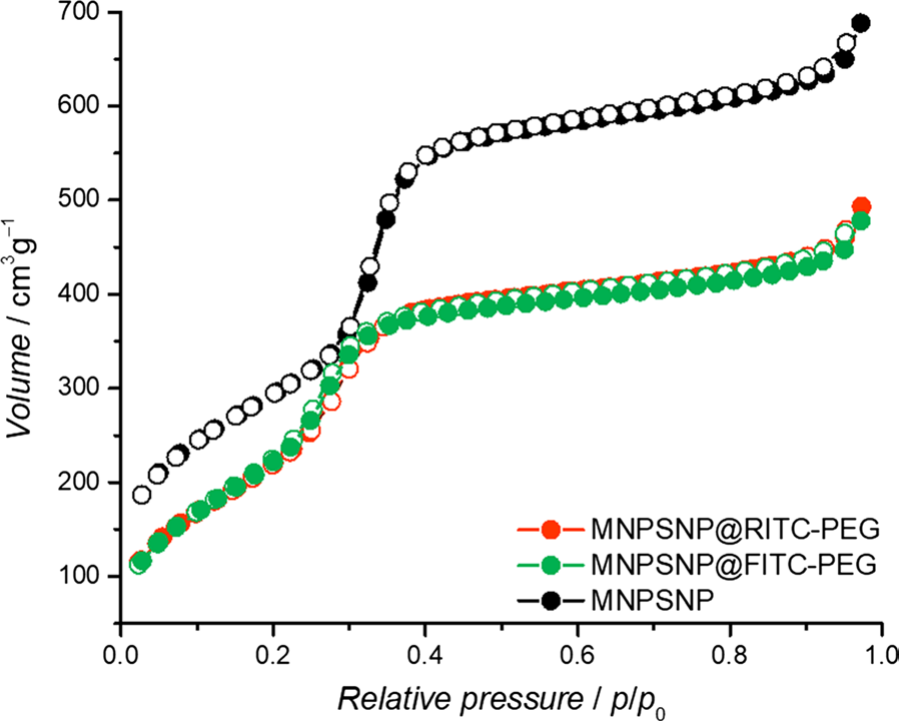
Nitrogen adsorption (dots) and desorption isotherm (circles) of unmodified MNPSNPs (black), MNPSNP@FITC-PEG (green) and MNPSNP@RITC-PEG (orange)

All spectra have a characteristic band of Fe_3_O_4_ at 576 cm^−1^ which corresponds to Fe–O stretching vibration modes [20]. At 1080 cm^−1^ an intensive Si–O–Si absorption is observed. Additionally to the vibrations of the magnetite cores and silica matrix, in the spectra of dye- and PEG-modified MNPSNPs, new modes between 2983 and 2897 cm^−1^ are visible. These vibrations indicate asymmetric and symmetric stretching vibrations of methylene groups of the additional organic compounds. The broad band in the range of 3750 cm^−1^ and 2800 cm^−1^ results of O–H stretching vibrations of surface silanol groups and water molecules, which are hydrogen-bonded between each other. N_2_ physisorption data showed for all samples type IV isotherms, which are typical for well-developed mesoporous structures (Fig. 6) [21].

Unmodified MNPSNPs have high porosity with a BET surface area of 1080 m^2^ g^−1^, a large total pore volume of 1.0 cm^3^ g^−1^ and a pore diameter of 3.9 nm, which was determined by the DFT method. After modification of MNPSNPs with FITC or RITC and PEG via post-grafting, the parameters for porosity were decreased due to the covalent attachment of organic groups on the silica matrix [22]. MNPSNP@FITC-PEG showed BET surface area, pore volume and pore diameter of 850 m^2^ g^−1^, 0.7 cm^3^ g^−1^ and 3.5 nm, respectively. MNPSNP@RITC-PEG had similar results with 875 m^2^ g^−1^ for BET surface area, 0.8 cm^3^ g^−1^ for pore volume and 3.7 nm for pore diameter.

**Fig. 7:**
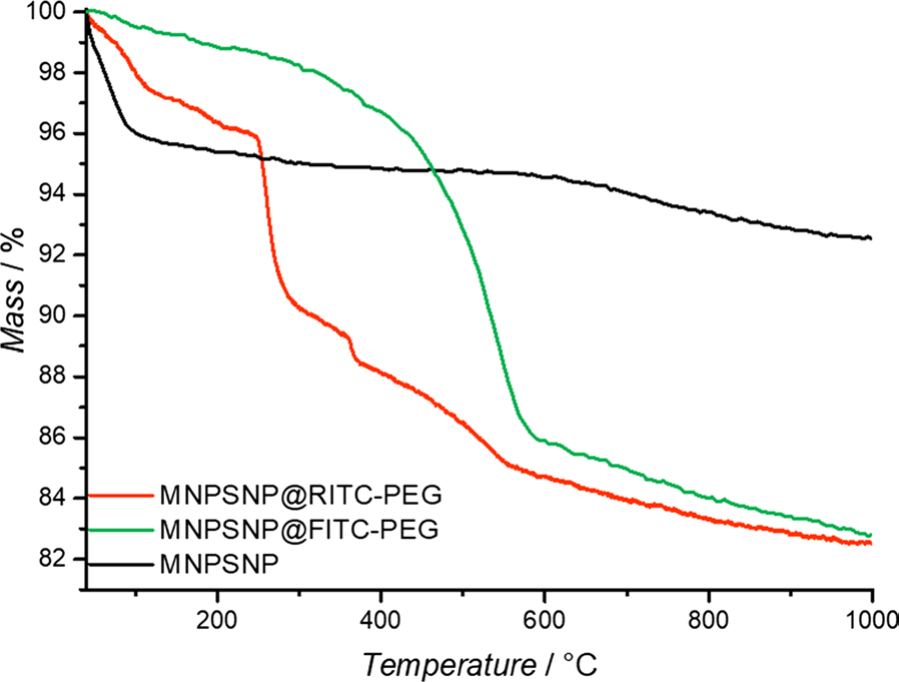
Thermogravimetric curves of unmodified MNPSNPs (black), MNPSNP@FITC-PEG (green) and MNPSNP@RITC-PEG (orange)

The modification with dye and PEG could also be verified by TGA (Fig. 7). Compared to unmodified MNPSNPs with a weight loss of 1.8% in a temperature range between 120 and 700 °C based on a dehydroxylated silica surface, FITC- and PEG-modified MNPSNPs showed a weight loss of 14.5% while the weight loss for RITC- and PEG-modified MNPSNPs was 13.5%.

### Cell proliferation

**Fig. 8:**
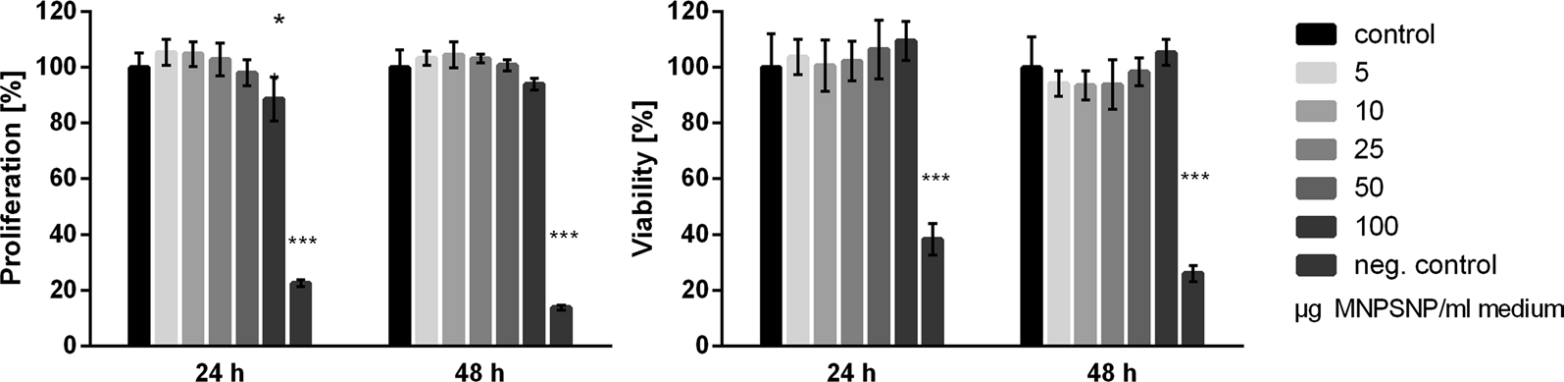
Cell proliferation and cell viability after 24 and 48 h of NIH-3T3 cells. Control group (0 µg MNPSNPs) compared to concentrations of 5, 10, 25, 50 and 100 µg MNPSNPs/mL cell culture medium. Negative controls include 5% DMSO. Mean ± SD, *p < 0.05, ***p < 0.001, n = 6

**Fig. 9:**
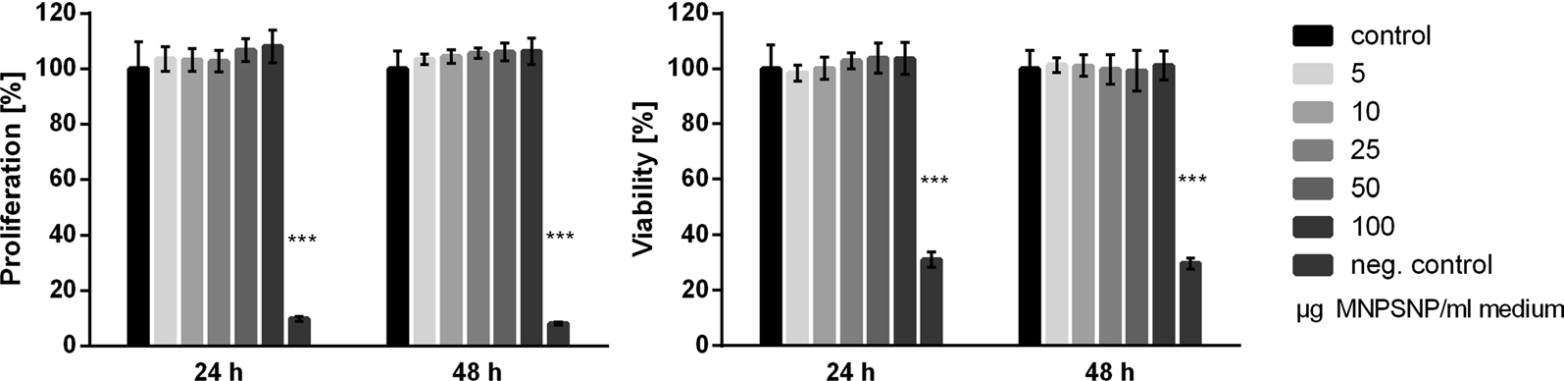
Cell proliferation and cell viability after 24 and 48 h of HepG2 cells. Control group (0 µg MNPSNPs) compared to concentrations of 5, 10, 25, 50 and 100 µg MNPSNPs/mL cell culture medium. Negative controls include 5% DMSO. Mean ± SD, *p < 0.05, ***p < 0.001, n = 6

Cell proliferation was significantly reduced for the NIH-3T3 cells at a concentration of 100 µg MNPSNP/ml cell culture medium at 24 h (*p < 0.05) but not for other time points or concentrations (Fig. 8). For the HepG2 cells there was no statistical significance shown for cell proliferation at any time point (Fig. 9).

### Cell viability

Cell viability was not influenced by the MNPSNPs for 24 and 48 h for both NIH-3T3 (Fig. 8) and HepG2 cell lines (Fig. 9).

### Accumulation of MNPSNPs at different implant materials in vitro

**Fig. 10:**
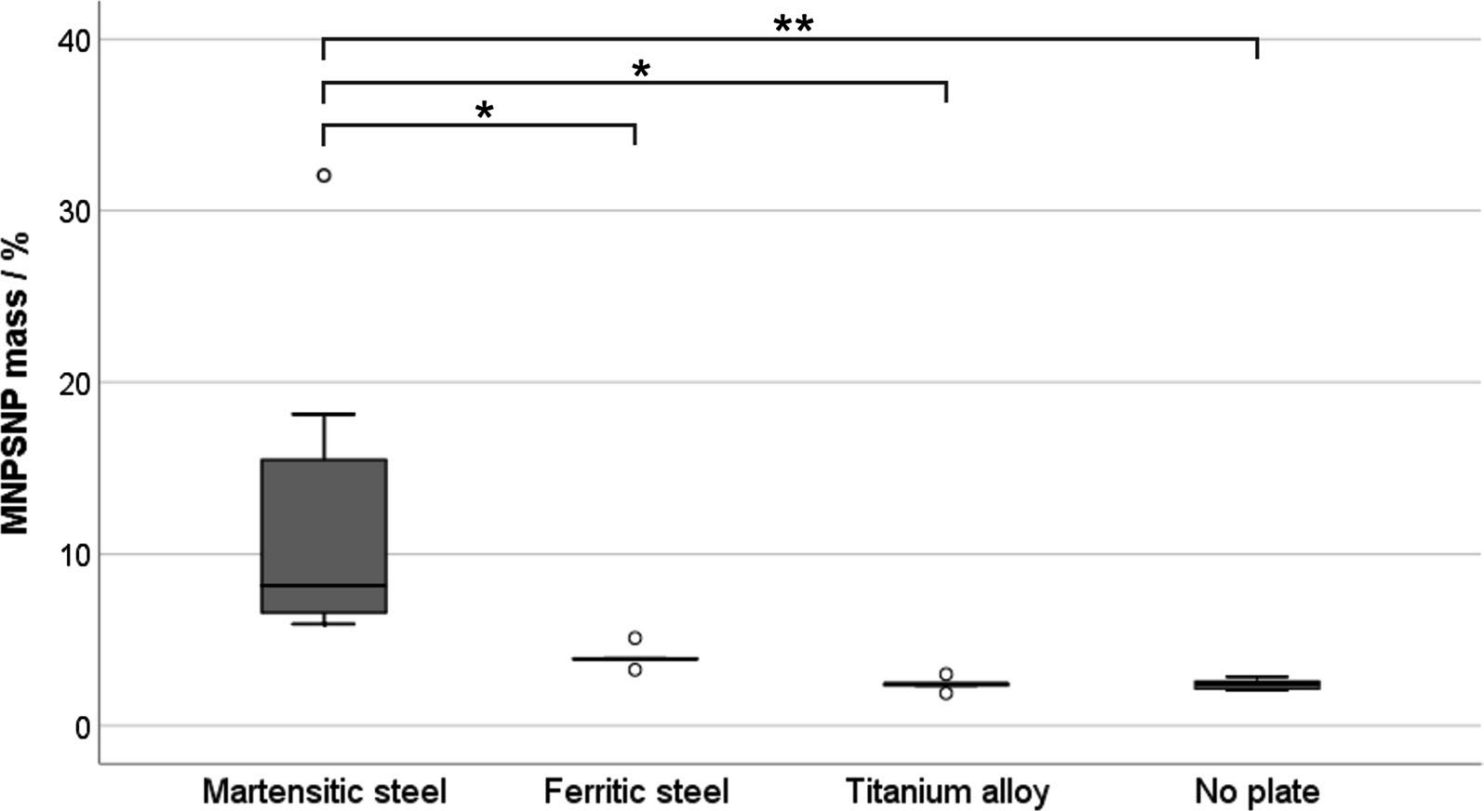
Percentage in vitro accumulation of MNPSNPs at different implant materials (*M* martensitic steel, *F* ferritic steel, *T* titanium alloy) and in the magnetic field without plate (*N* no plate in tube systems), *p < 0.05, **p < 0.01

The martensitic plates were able to retain significantly more MNPSNPs in the in vitro set up than the ferritic plates (p = 0.041) and both controls (T: p = 0.013; N: p = 0.006) (Fig. 10). At ferritic plates the amount of accumulated MNPSNPs did neither differ significantly to the titanium alloy plate (p = 0.963) nor to the control without plate (p = 0.952).

**Fig. 11:**
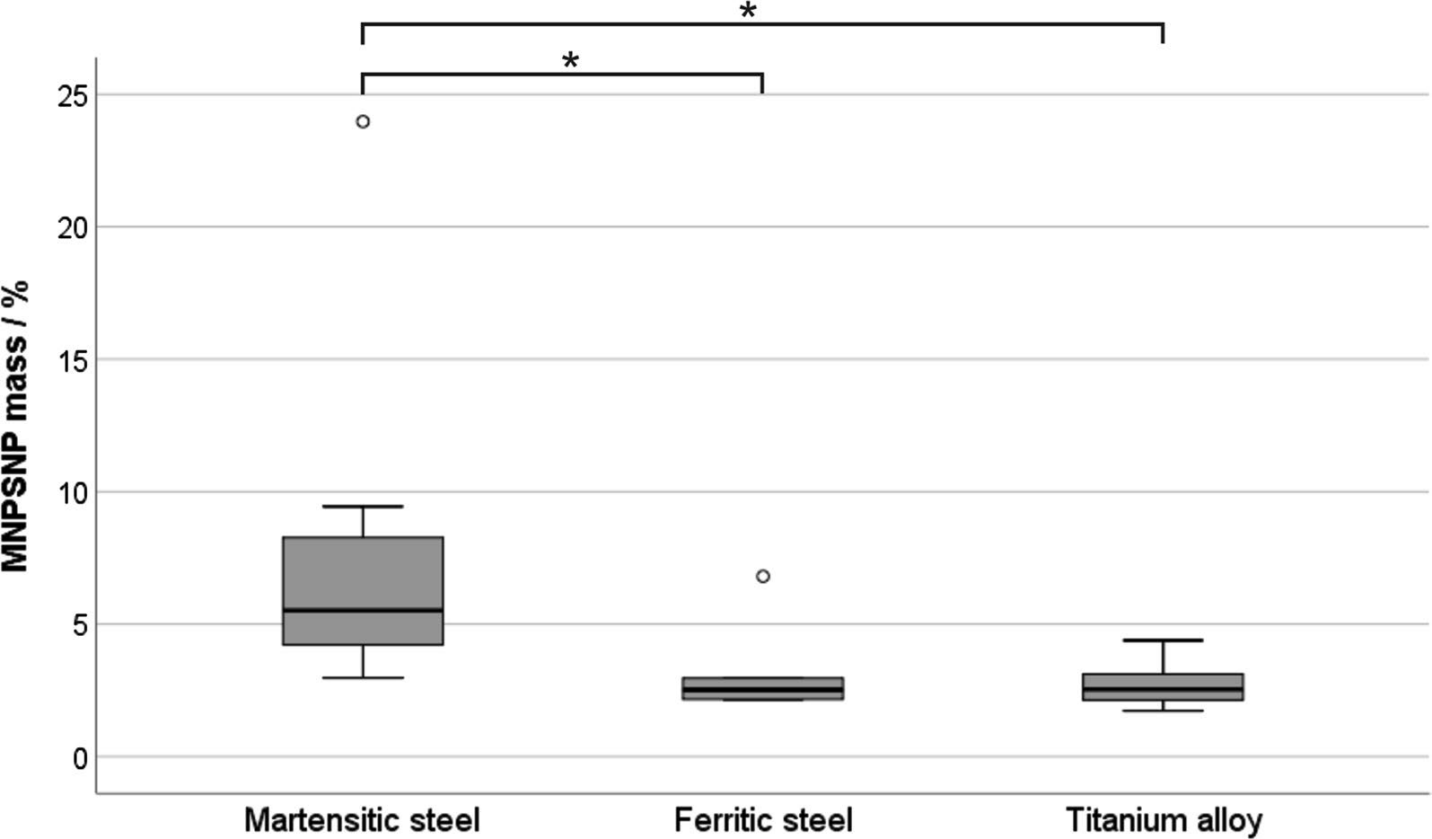
Percentage MNPSNP mass at the different plates after in vitro accumulation of the MNPSNP suspension in the magnetic field and subsequent 3 min additional circulation time, *p < 0.05

Remanent properties of martensitic steel plates could be shown by the higher percentage amount of MNPSNPs after 3 min circulation time without external magnetic field. Compared to the loading suspension, martensitic plates accumulated a median of 5.5% MNPSNPs (min: 3%, max: 23.9%) while ferritic plates (median 2.5%, min: 2.1%, max: 6.8%, p = 0.019) and titanium alloy plates (median: 2.5%, min: 1.7%, max: 4.4%, p = 0.015) accumulated only about half of that amount. Ferritic steel and titanium showed similar values (p = 0.715). Results are depicted in Fig. 11.

### In vivo set up

All mice showed no adverse effects around the plates during the post-surgical follow up. After euthanasia plates could be easily extracted from the subcutis.

#### MNPSNPs detection in tissues by fluorescence analysis and pathological changes of organs

In neither organ smear samples nor blood samples MNPSNPs could be detected via fluorescence analysis.

**Fig. 12:**
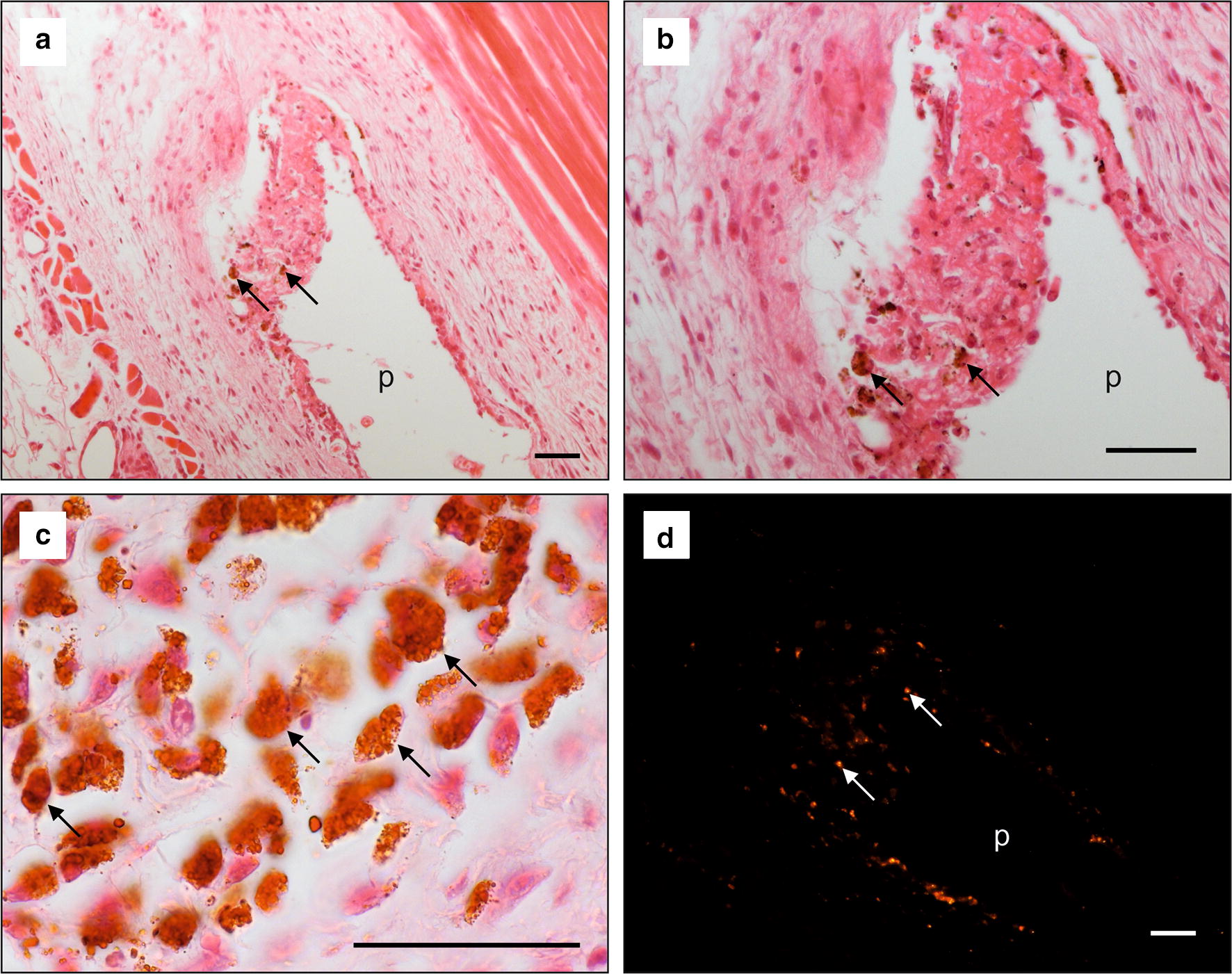
**a**–**c** H.E. staining of the skin-muscle-layer with former plate location (p) showing macrophage infiltration, fibrotic tissue and MNPSNPs associated to cells (black arrows); **d** Fluorescent MNPSNPs (orange spots, white arrows) in the fibrotic tissue. All scale bars: 50 µm

Histologic slices of skin and muscles showed low-grade fibrosis with histiocytic infiltration and fluorescent particles around the former plate location. These small MNPSNP clusters were either diffusely distributed or found in cell-like structures (Fig. 12).

**Fig. 13:**
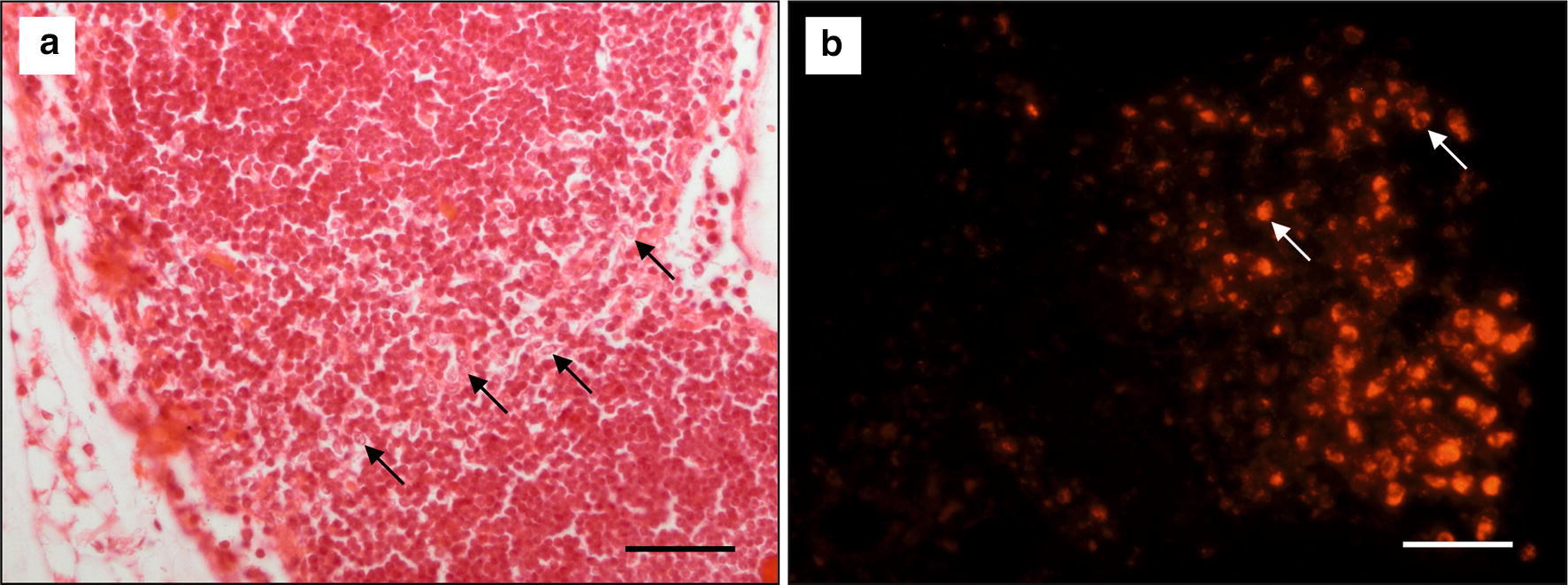
**a** H.E. staining of the *Ln. iliacus* with histiocytic inclusions (black arrows); **b** Fluorescent MNPSNPs (orange spots) in a corresponding area associated to cells (white arrows). Scale bars: 50 µm

Corresponding to the organ smear samples, fluorescence analysis of the histologic slices of the organs also showed no MNPSNPs except for one animal in which one lymph node showed fluorescent particles of different density exclusively in the margin sinus and para cortex (Fig. 13b). These particles were associated with roundly shaped cells.

In the corresponding H.E. slice it could be shown that the fluorescence was associated to macrophages. This lymph node also showed histiocytic inclusions in the margin sinus and para cortex (Fig. 13a). No other pathological changes could be found in any other slice.

#### MNPSNP detection on explanted plates by fluorescence analysis

**Fig. 14:**
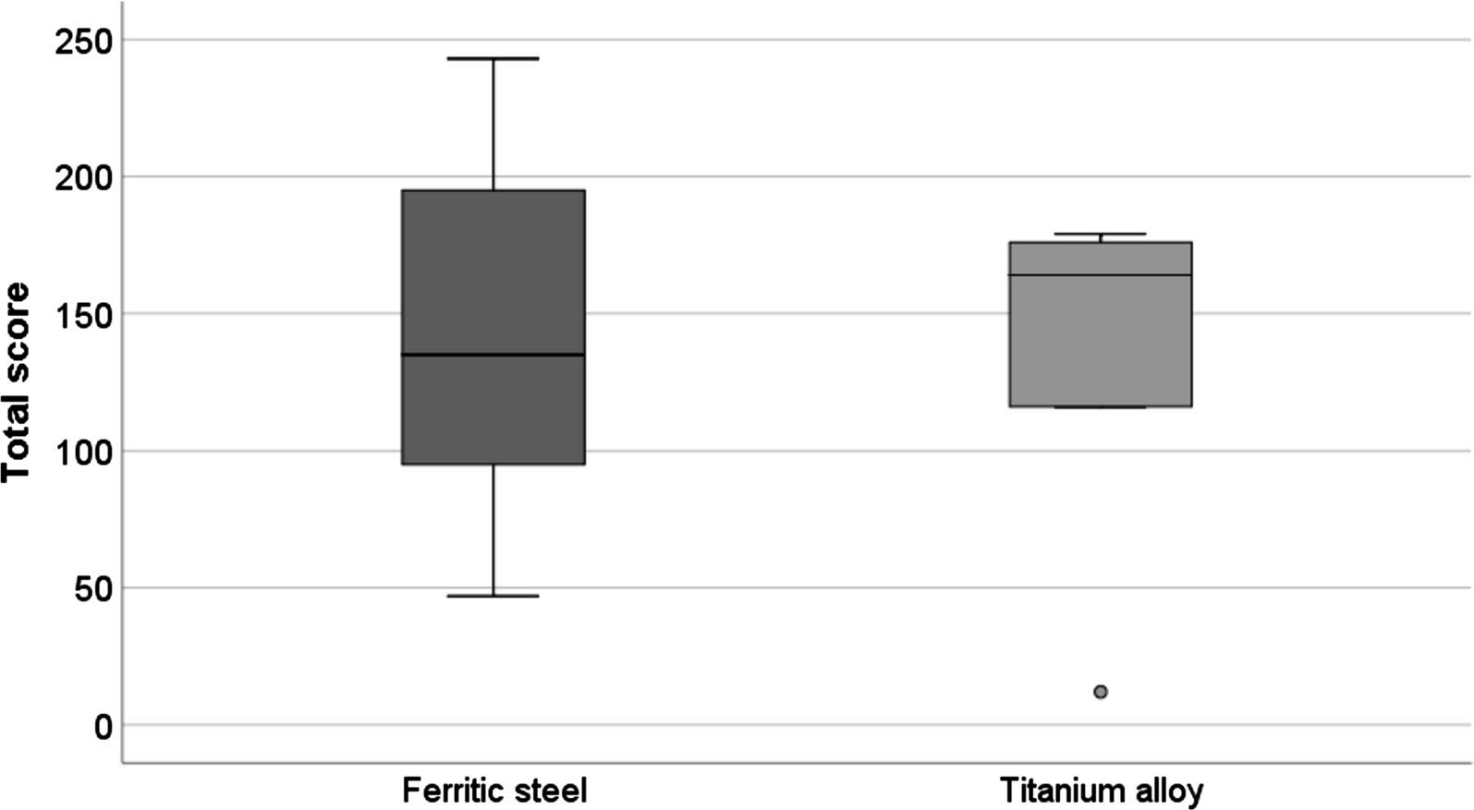
Summed score for evaluation of droplets containing MNPSNPs on explanted ferritic steel and titanium alloy plates 1 week after in vivo injection subcutaneously near the implant

Droplet samples of explanted ferritic steel and titanium alloy plates showed no differences in the remaining MNPSNP mass 1 week after subcutaneous injection. On both implants a mean total score of 143 ± 77 and 129 ± 70 for ferritic and titanium plates, respectively, were detectable in the suspension after ultrasonic treatment of the implants (Fig. 14).

The evaluation of the plates with fluorescence microscopy showed individual to larger conglomeration of nanoparticles randomly distributed on the surface of the plates. No differences between both materials could be found.

## Discussion

Implant-associated infections are a major challenge in the medical care of orthopedic surgery. The treatment of such inflammation processes is often based on the routine administration of antibiotics or the application of antimicrobial implant coatings. But especially the selective treatment of acute infections is very difficult due to an insufficient agent accumulation in the target tissue. For the successful treatment, excessive drug levels may be indispensable to reach adequate concentrations in the infection area. Consequently, the risk of undesirable side effects increases while the success of the therapy is not guaranteed [23]. In the present study, the implant-directed magnetic drug targeting (ID-MDT) has a great potential for the improvement of the treatment of implant-related infections. Here, the utilization of multifunctional magnetic nanoporous silica nanoparticles (MNPSNPs) as carriers for pharmaceuticals or oligonucleotides and growth factors enables the accumulation in selective areas of the organism in combination with magnetizable implants and an externally applied magnetic field. The MNPSNPs were composed of superparamagnetic Fe_3_O_4_ NPs, which were encapsulated in a nanoporous silica shell. The silica shell was functionalized with PEG and organic fluorophores like FITC or RITC.

For the realization of ID-MDT and thus the improvement of the selective treatment of implant-associated infections a core–shell nanoparticle system with several challenges is required. The first very important component is the magnetic core material. Due to their good magnetic properties and the biocompatibility, the mostly used magnetic nanoparticles are magnetite (Fe_3_O_4_) or maghemite (γ-Fe_2_O_3_) [24]. For the synthesis of the oleic-acid capped Fe_3_O_4_ NPs in this work the so-called Massart method was used, which is the most popular procedure based on a coprecipitation of Fe^2+^ and Fe^3+^ salts with the addition of a base in inert atmosphere [25]. In this method the particle size can be adjusted by the variation of temperature, pH or the Fe^2+^/Fe^3+^ ratio. The particles size is a key factor for the utilization of magnetic nanoparticles in biomedical applications. Particle sizes with diameters of 10–100 nm are preferred for in vivo applications because the danger of rapid renal clearance is reduced, which occurs for NPs smaller than 10 nm. For NPs > 200 nm an internalization by the reticuloendothelial system (RES) is possible, resulting in an accumulation of particles in the liver and the spleen before a loaded drug can be delivered to the target tissue [26]. Directly related to the particle size in magnetic materials is the magnetic behavior. While Fe_3_O_4_ as bulk material is ferrimagnetic, Fe_3_O_4_ NPs are superparamagnetic. Here the nanometer-sized crystals can be considered as “single-domain” or “magnetic domain” [27]. In comparison to bulk material with a ferrimagnetic behavior, superparamagnetic Fe_3_O_4_ NPs have no remnant magnetization after the removal of an external magnetic field (EMF) [28]. As a result, the risk of particle aggregation based on remaining residual magnetization is reduced. Fe_3_O_4_ NPs with this type of magnetism are also called SPIONs (superparamagnetic iron oxide nanoparticles). They should have particle sizes under 30 nm for this magnetic phenomenon [29]. However, Fe_3_O_4_ NPs with particles sizes under 10 nm show only low values of saturation magnetization. The saturation magnetization is the maximum value of magnetization which can be reached after the application of an EMF. Too small saturation magnetization could be critically for the successful accumulation of MNPSNPs on a magnetizable implant in combination with an EMF, especially when taking into account the presence of the additional nanoporous silica shell around the core material. The Fe_3_O_4_ NPs were coated with oleic acid as stabilizing ligand to prevent early particle agglomeration in suspensions [30]. The oleic acid-capped Fe_3_O_4_ NPs used in this study showed particles sizes of 10 ± 2 nm and superparamagnetic behavior with no remanence after the removal of an EMF and a saturation magnetization of 48 emu g^−1^ and thus excellent properties as magnetic core material for the MNPSNPs with regard to the ID-MDT.

The superparamagnetic core particles were next equipped with a nanoporous silica shell to yield MNPSNPs. Since their discovery in 1992 [31], nanoporous silica materials have been researched as nanoparticles or as implant surface coatings [8, 32–36]. These materials combine the advantages of an excellent biocompatibility, high specific surface areas, large pore volumes and adjustable pore sizes with narrow pore size distributions as well as facile chemical modification using silanization reactions. Thus, they have a great potential for loading with drugs, proteins or growth factors [10, 37, 38]. As presented in the TEM image the MNPSNPs were spherical core–shell nanoparticles with Fe_3_O_4_ as core material and main particles sizes of approximately 110 nm. Especially for the use of the MNPSNPs in biomedical applications the particle size should be < 200 nm to prevent an early internalization by the RES, as previously mentioned. N_2_ physisorption measurements confirmed a high BET surface area and a large pore volume and thus very high porosity for potential drug delivery. Investigations of the MNPSNPs with the VSM showed a clear decrease of the saturation magnetization in comparison to the Fe_3_O_4_ NPs. Due to the nanoporous silica shell around the cores, the amount of magnetic material is markedly smaller in comparison to the pure oleic acid-capped Fe_3_O_4_ NPs. Nevertheless, the MNPSNPs still have superparamagnetic behavior with no remanence after the removal of an EMF, which is one of the most important aspects with regard to the application in ID-MDT.

Due to the presence of silanol groups on their surface, nanoporous silica materials have a versatile surface chemistry. These groups can be used for the coupling of other silanes and thus the functionalization with a wide variety of functional groups [39, 40].

The MNPSNPs in this study were functionalized with the organic fluorescent dyes FITC or RITC for in vitro and in vivo investigations. FITC and RITC are the derivatives of fluorescein and rhodamine B and due to their fluorescent properties they are widely used compounds for bioimaging applications [19, 41]. Here the dyes were modified in a first step via APTMS to FITC-APTMS or RITC-APTMS. Afterwards these thioureas were coupled with the post-grafting method on the silica surface.

In addition to the attachment of fluorophores to the surface of MNPSNPs, the coupling of PEG to the silica system was a further functionalization in this study. This so-called PEGylation is a versatile method to improve dispersibility as well as biocompatibility and biodistribution due to the depression of the nonspecific binding of PEGylated nanoparticles to blood proteins and macrophages [42–44]. Consequently, with this promising approach the particles have an increasing hydrophilicity, a lower decomposition rate and thus a higher blood circulation time. TEM images of the modified MNPSNPs showed no loss of spherical shape or an attack of the surface. The mean particles sizes of MNPSNP@FITC-PEG and MNPSNP@RITC-PEG were similar in comparison to the unmodified MNPSNPs. Thus, the characteristic composite structure of the MNPSNPs was preserved. In comparison to the particles sizes observed transmission electron micrographs, DLS showed larger particle diameters due to the presence of a hydration shell layer in aqueous media. Due to the modification with the dye and the PEG residues, the modified MNPSNPs showed an increase of particle diameter compared to unmodified particles. Nevertheless, the narrow particles size distributions as well as the strong negative zeta potentials indicate colloidal stability. The successful functionalization of the MNPSNPs with the organic fluorophores and PEG was confirmed via FT-IR spectroscopy as well as thermogravimetric analysis and played a key role in the assessment of MNPSNP accumulation and distribution in the presented examinations. Investigations with N_2_ physisorption presented a small decrease of BET surface areas, pore volumes and pore sizes, i.e. the covalent attachment of organic groups on the nanoporous silica surface led to a minimal loss of porosity. Nevertheless, MNPSNP@FITC-PEG and MNPSNP@RITC-PEG are still highly porous and therefore offer great potential for the loading and delivery of drugs or other cargos.

The viability and proliferation of NIH-3T3 and GepG2 cells were examined after incubation with MNPSNPs to assess the in vitro cytotoxicity as recommended, e.g., by Brunner et al. [45]. Both chosen cell lines are common to test different types of nanoparticles for their cytotoxicity [46–49]. In our study, neither viability nor proliferation of both cell lines was influenced by any concentration of MNPSNPs/mL medium except for one. At the highest concentration of 100 µg MNPSNPs/mL medium the cell proliferation of NIH-3T3 cells was decreased after 24 h, while the cell viability stayed on the same level as the control group. This could be a sign for an increase in cell activity at high MNPSNP concentration and dying of some cells due to the MNPSNP-induced stress. Probably, this is due to the “overload dose” or “extreme dose” effect, where high doses of nanoparticles only act physically as a barrier for diffusion around the cells, thus blocking access of nutrient solution and dissolved gases like oxygen [50]. This does not indicate a specific cytotoxic effect. After 48 h the cell proliferation was back on a level similar to the control group which indicates their recovery within a 24 h period. Zasonska et al. examined the in vitro cytotoxicity of silica core–shell nanoparticles on lymphocytes which have a similar structure as the MNPSNPs used in the present study and assessed their nanoparticles as only lowly cytotoxic [51]. Also Jang et al. examined iron/silica core/shell nanoparticles with and without PEGylation by MTT assay on a HeLa cell line and found that the PEGylation enhanced the cell viability to a maximum of 90% depending on the concentration used [52]. They concluded that PEGylated nanoparticles are excellent candidates for in vivo use. Regarding these studies and the results that MNPSNP treatment showed no cytotoxic effects in vitro to a mouse fibroblast and a human hepatoma cell line in our own study, they can be considered safe for in vivo use.

The martensitic material led to a significantly higher accumulation of MNPSNPs within a circular tube system compared to a paramagnetic control plate (titanium alloy) or the magnetic field alone. These results confirm the conclusions of a previous examination on this topic [14]. In comparison to the here present study, Angrisani et al. used the same martensitic steel and similar nanoporous silica nanoparticles, but the external magnetic field was produced by permanent neodymium magnets with a considerable lower strength. In the first instance they showed that, as hypothesized, the magnetizable material led to an increased magnetic field strength and, further, that the martensitic plate was able to retain a significantly higher quantity of nanoporous silica nanoparticles than the magnetic field of the neodymium magnets alone. This result was attributed to the intensified magnetic field strength. The assessment of the effect of an external magnetic field on magnetic drug targeting has changed over the last years. Hofmann-Amtenbrink et al. mentioned in 2009 that directing magnetic nanoparticles to any cell, tissue or tumor in the body was unrealistic as the magnetic force is not high enough to guide the particles through the blood system [53]. Hournkumnuard and Natenapit determined by simulation, that an externally applied magnetic field strength of not greater than 0.8 T significantly improved the effectiveness of concentrating ferromagnetic drug carrier nanoparticles around a ferromagnetic target microwire within a small vein [54]. Considering these facts and following the other part of our project, we worked with a strong electromagnet, which induced a magnetic field of around 1.8 T on the mouse skin during the application. Additionally, inside the aimed region the magnetic field was even higher because of the internally intensifying implant. However, it was unexpected, that ferritic steel plates did not accumulate more MNPSNPs than the controls, particularly with regard to their high permeability. This result has to be clarified in further examinations. In context of the above mentioned references we can conclude that the martensitic material influences the MNPSNP accumulation in the required way and therefore may be a promising candidate for an in vivo application. However, although well biocompatible, martensitic as well as ferritic plates are not approved for clinical use, so future investigation will be needed about the transfer either on approved materials or the approval of new ones with the required properties.

As expected, martensitic plates showed their higher remanence by the higher percentage of retained MNPSNPs after additional circulation time without external magnetic field. Angrisani et al. also showed the remanent behavior of the martensitic steel 1.4122. In their experimental set up with no fluid cycle, the plate was placed outside the fluid flow and the nanoparticle suspension passed the location of the plate only once. Still the martensitic plate was able to inhibit the clearance of the accumulated nanoparticles by nanoparticle-free fluid flow after the permanent magnets and therewith the external magnetic field were removed [14]. Although ferritic materials with their higher permeability should lead to an improved initial accumulation through their stronger enhancement of the external magnetic field, this effect is immediately abandoned after disabling of the magnetic force. Here, remanent implants may further improve the advantage of a magnetizable implant material as they would provide a maintaining magnetic effect. On the one hand, this could further attract MNPSNPs that so far had not been accumulated; on the other hand, the force exerted on the superparamagnetic nanoparticles is stronger and longer-lasting which could possibly support the invasion of MNPSNPs into an existing biofilm. These considerations must be proved in future studies.

The in vitro tube system only simulates the flow velocity of small blood vessels, but there are many more factors and forces influencing the fate of nanoparticles in vivo. Biodistribution and cellular uptake of different kind of nanoparticles after intravenous administration either for drug targeting purpose or as MRI contrast agent, among others, is frequently described in literature. Mohammed reviewed that “MNPs surface interact with immune system, extracellular matrices, plasma proteins and non-targeted cells” [55]. Particles would be filtered out of blood by the first capillary bed they traverse, especially but mostly temporarily by the lung. Depending on size, shape, coatings and other properties, cells of the mononuclear phagocyte system (MPS) in the liver (highest percentage), spleen, lungs, bone marrow and partly in kidney would rapidly engulf most particles and inhibit reaching the target tissue or region [12, 27, 55–57]. Nevertheless, there is a lack of literature dealing with MNP-attracting magnetic implants as active target inside the body. We will evaluate the biodistribution of our MNPSNPs after intravenous application in mice in another part of this project. In this paper, we focused on subcutaneous administration as a preliminary experiment to examine nanoparticle-implant-interaction with external applied magnetic field locally in the surrounding tissue.

Our histological examinations of the tissue area where the implant had been located showed a large part of locally infiltrated nanoparticles associated to cells, likely dendritic cells, macrophages or fibroblasts. The MNPSNPs were either on the cell surfaces or in the cytoplasm, possibly both. The same association was found in one lymph node. Literature related to immunological reactions also reported comparable situations. In principal, aggregated nanoparticles representing foreign material would be phagocytosed by migrated neutrophils and macrophages or by macrophages and dendritic cells of corresponding lymph nodes [58]. Regarding lymphatic involvement, Hawley et al. and Saraf et al. described the uptake of different colloids in the lymph nodes after subcutaneous administration [59, 60]. The lymphatic uptake constitutes an opportunity for nanoparticles as a drug delivery system for lymph targeting and is used for example in tumor diagnosis and treatment [61]. In the present study, one of ten lymph nodes showed characteristic fluorescence of MNPSNPs. There are different factors which could have led to this ratio. They range from individually varying drainage areas of lymph nodes through to examination time-dependent factors. A higher or lower interstitial resistance to molecular transport because of a slightly deeper or more superficial injection of MNPSNPs, or MNPSNP clusters trapped in the interstitial space for prolonged times according to their size (> a few 100 nm) could lead to a delayed transportation of MNPSNPs to the medial iliac lymph node [60, 62]. Examinations at a posterior time point possibly could show more pronounced MNPSNP accumulation in lymph nodes. On the contrary, an increasing lymphatic drainage for instance due to excessive body care by individual mice with the tongue would lead to an earlier accumulation of MNPSNPs in the local lymph node [63]. In addition, the distribution of the MNPSNPs within the lymph node is inhomogenous which led to slice-dependent quantities of fluorescent cells. A final statement on the lymphatic distribution of MNPSNPs would require a complete histological processing of all relevant lymph nodes.

Considering all these arguments from production of the MNPSNPs to their first usage in vivo, more research is needed before advancing to clinical application in the future. For the treatment of implant-associated infections by ID-MDT, the MNPSNPs will be loaded with antibiotics and administration of these loaded particles should only be performed when an implant infection exists. The long-term objective is to create a drug release kinetic which ensures a continuous release over quite a few days. Most beneficial for the patient would be the successful accumulation of MNPSNPs on the implant due to its magnetization after a single dose injection and one magnetic field application. However, if necessary, repeated treatments are conceivable. With further surface modifications of the MNPSNPs a delayed and moreover stimuli-responsive release is possible to guarantee the antibiotic effect only in the target area as well as a demand-adapted circulation time.

## Conclusion

MNPSNPs showing superparamagnetic properties could be synthesized and functionalized with fluorescent dyes (FITC, RITC) and PEG successfully and reproducibly. In vitro proliferation and viability tests showed good biocompatibility and predicted safe in vivo use. Plates of different magnetic properties influenced the accumulation of MNPSNPs in vitro; martensitic steel 1.4112 showed the highest accumulation and retention of MNPSNPs in a circular tube system. In vivo, the nanoparticles were well biocompatible after s.c. injection in the mouse. They were found at the implant surface as well as in the surrounding tissue and—partly—the local lymph node. All these results favour a prospective use of MNPSNPs in ID-MDT. Since reaching various localizations in the body via the blood system is the long-term objective of ID-MDT, examinations on biodistribution and accumulation after intravenous injection will have to be performed next. The loading and controlled releasing of antibiotics as well as the circulation time of MNPSNPs will be adapted by surface modifications. Finally, the evaluation of its efficiency will be performed in different infection models.

After successful completing of these examinations and together with an ideally designed implant this concept will offer a gentle but most efficient way to treat implant-associated infections at any localization in the organism avoiding revision surgeries and minimizing the development of antibiotic resistances.

## Additional file

**Additional file 1.** Additional figures and table.

## Abbreviations

MNPSNPs: magnetic nanoporous silica nanoparticles
ID-MDT: implant-directed magnetic drug targeting
e.g.: exempli gratia
et al.: et alii
NPs: nanoparticles
PEG: poly(ethylene) glycol
FITC: fluorescein isothiocyanate
RITC: rhodamine B isothiocyanate
CTAB: cetyltrimethylammonium bromide
TEOS: tetraethyl orthosilicate
APTMS: 3-aminopropyl trimethoxysilane
MW: molecular weight
Fe_3_O_4_: magnetite
min: minute
h: hour
sec: second
TEM: transmission electron microscopy
FT-IR: Fourier-transform infrared spectroscopy
KBr: potassium bromide
N_2_: nitrogen
BET: Brunauer–Emmet–Teller
DFT: density functional theory
TGA: thermogravimetric analysis
VSM: vibrating sample magnetometer
DLS: dynamic light scattering
PDI: polydispersity index
DMSO: dimethylsulfoxide
PBS: phosphate buffered saline
M: martensitic material
F: ferritic material
T: titanium alloy Ti90Al6V4
A. dest.: destilled water
n: number
RT: room temperature
m: mass
c: mass concentration
V: volume
s.c.: subcutaneously
BW: Body weight
H.E.: Hematoxylin–eosin
neg.: negative
SD: standard deviation
p: plate location
RES: reticuloendothelial system
EMF: external magnetic field
SPIONs: superparamagnetic iron oxide nanoparticles
MRI: magnetic resonance imaging
MNPs: magnetic nanoparticles
MPS: mononuclear phagocyte system
