# Supplementary material for: In vitro and in vivo accumulation of magnetic nanoporous silica nanoparticles on implant materials with different magnetic properties
Source: J Nanobiotechnology. 2018 Nov 27;16:96. doi: 10.1186/s12951-018-0422-6 (PMC6258308; doi:10.1186/s12951-018-0422-6)
Supplement: Supplementary file 1 — Additional file 1. Additional figures and table. [file 12951_2018_422_MOESM1_ESM.docx]

**Additional Information**

**In vitro and in vivo accumulation of magnetic nanoporous silica nanoparticles on implant materials with different magnetic properties**

Hilke Catherina Janßen^1†^, Dawid Peter Warwas^2†^, David Dahlhaus^3^, Piriya Taptimthong^4^, Manfred Kietzmann^3^, Peter Behrens^2^, Janin Reifenrath^1^, Nina Angrisani^1^*

^1^Hannover Medical School, Clinic for Orthopedic Surgery, NIFE-Lower Saxony Centre for Biomedical Engineering, Implant Research and Development, Stadtfelddamm 34, 30625 Hannover

^2^Institute for Inorganic Chemistry, Leibniz University Hannover, Callinstraße 9, 30167 Hannover, Germany

^3^Institute of Pharmacology, Toxicology and Pharmacy, University of Veterinary, Medicine, Foundation, Bünteweg 17, 30559 Hannover, Germany

^4^Insitute of Micro Production Technology, Leibniz University Hannover, An der Universität 2, 30823 Garbsen

^†^Hilke Catherina Janßen and Dawid Peter Warwas contributed equally to this work

*Corresponding author

[Jessica.meissner@tiho-hannover.de](mailto:Jessica.meissner@tiho-hannover.de)

Hilke Catherina Janßen janssen.hilke@mh-hannover.de

Dawid Peter Warwas dawid.warwas@acb.uni-hannover.de

David Dahlhaus david.dahlhaus@tiho-hannover.de

Piriya Taptimthong taptimthong@impt.uni-hannover.de

Manfred Kietzmann manfred.kietzmann@tiho-hannover.de

Peter Behrens peter.behrens@acb.uni-hannover.de

Janin Reifenrath reifenrath.janin@mh-hannover.de

**
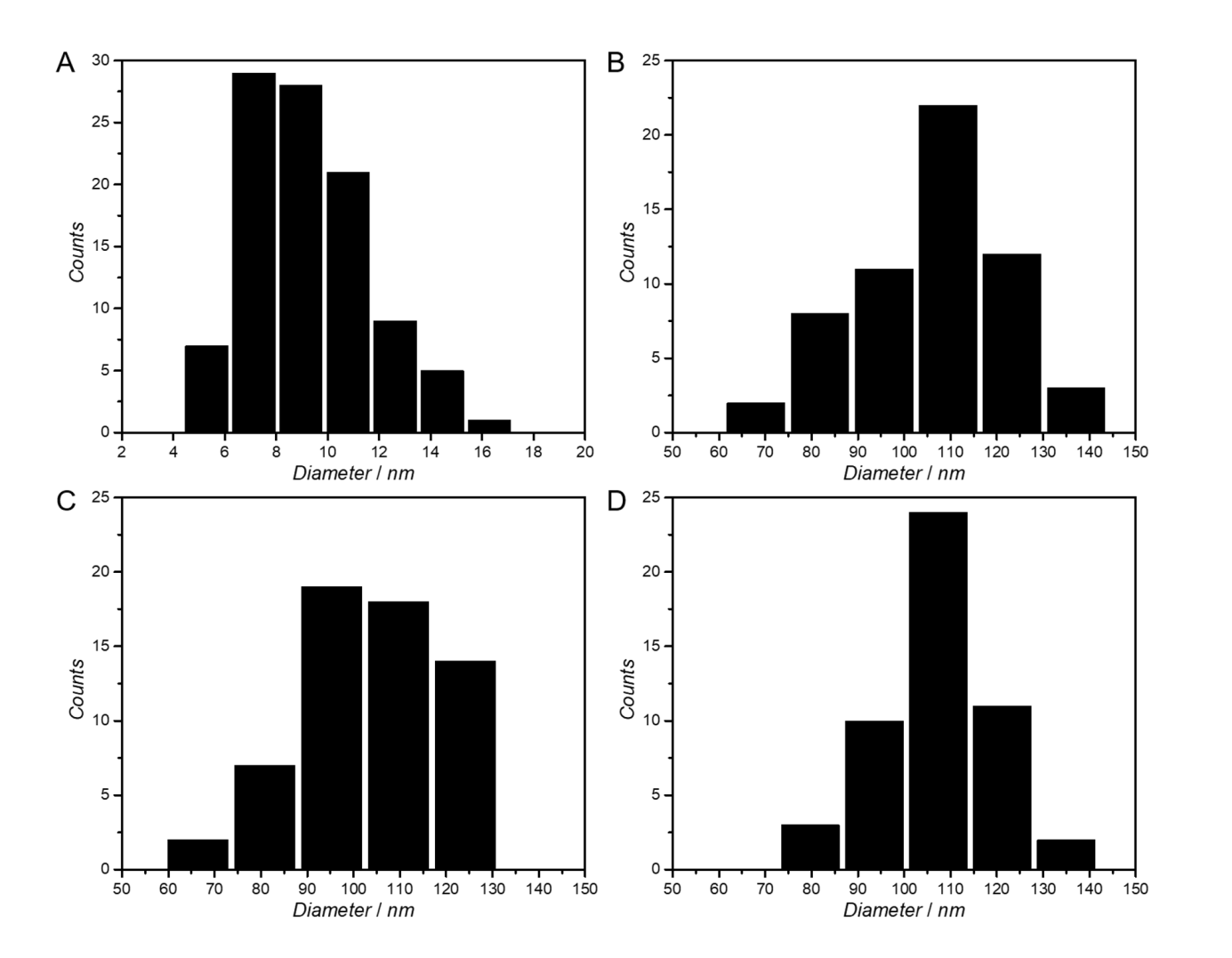
**

Figure S1: Particle size histograms of (A) oleic acid-capped Fe_3_O_4_ NPs, (B) unmodified MNPSNPs, (C) MNPSNP@FITC-PEG, (D) MNPSNP@RITC-PEG.


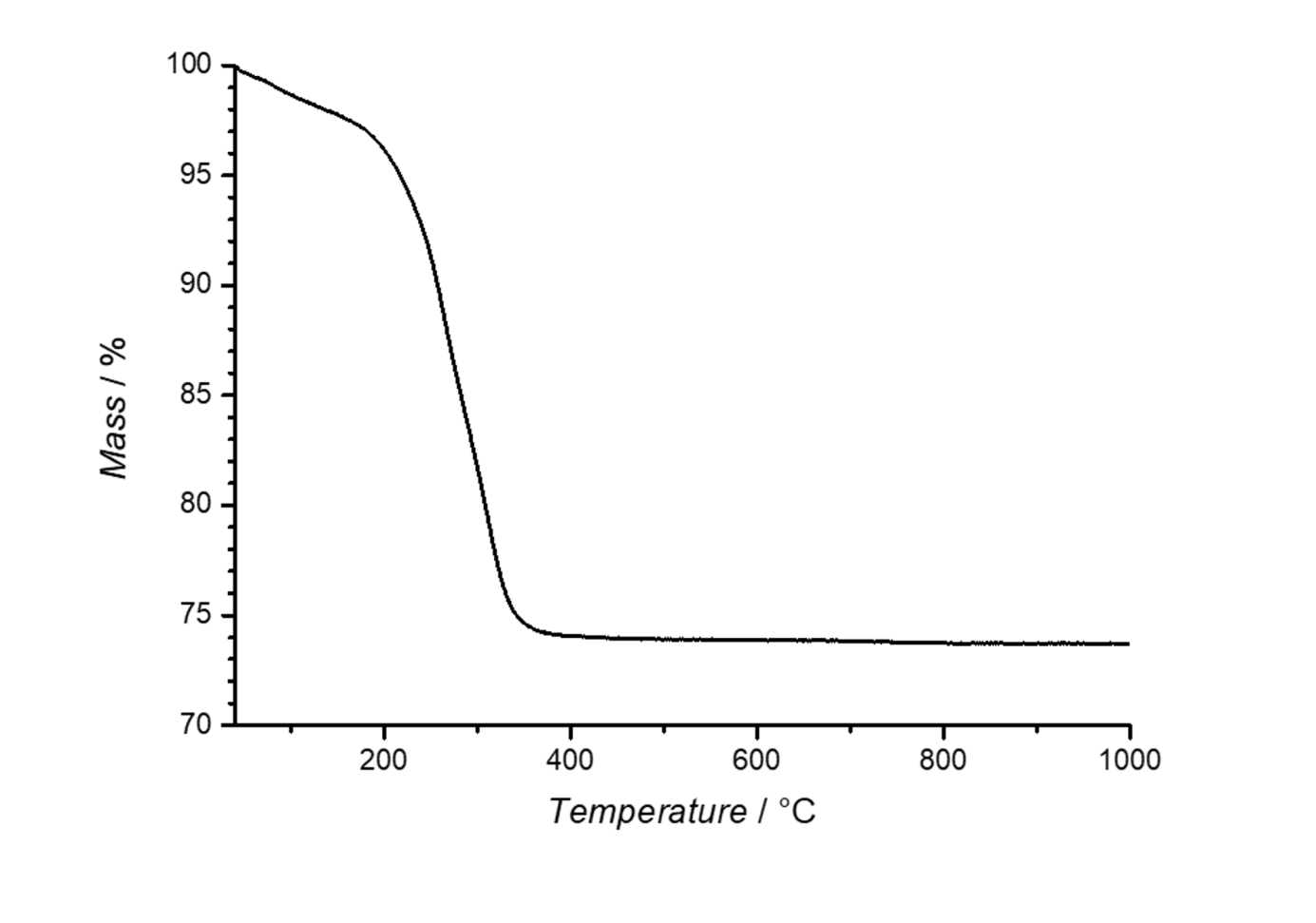


Figure S2: Thermogravimetric curve of oleic acid-capped Fe_3_O_4_ NPs.


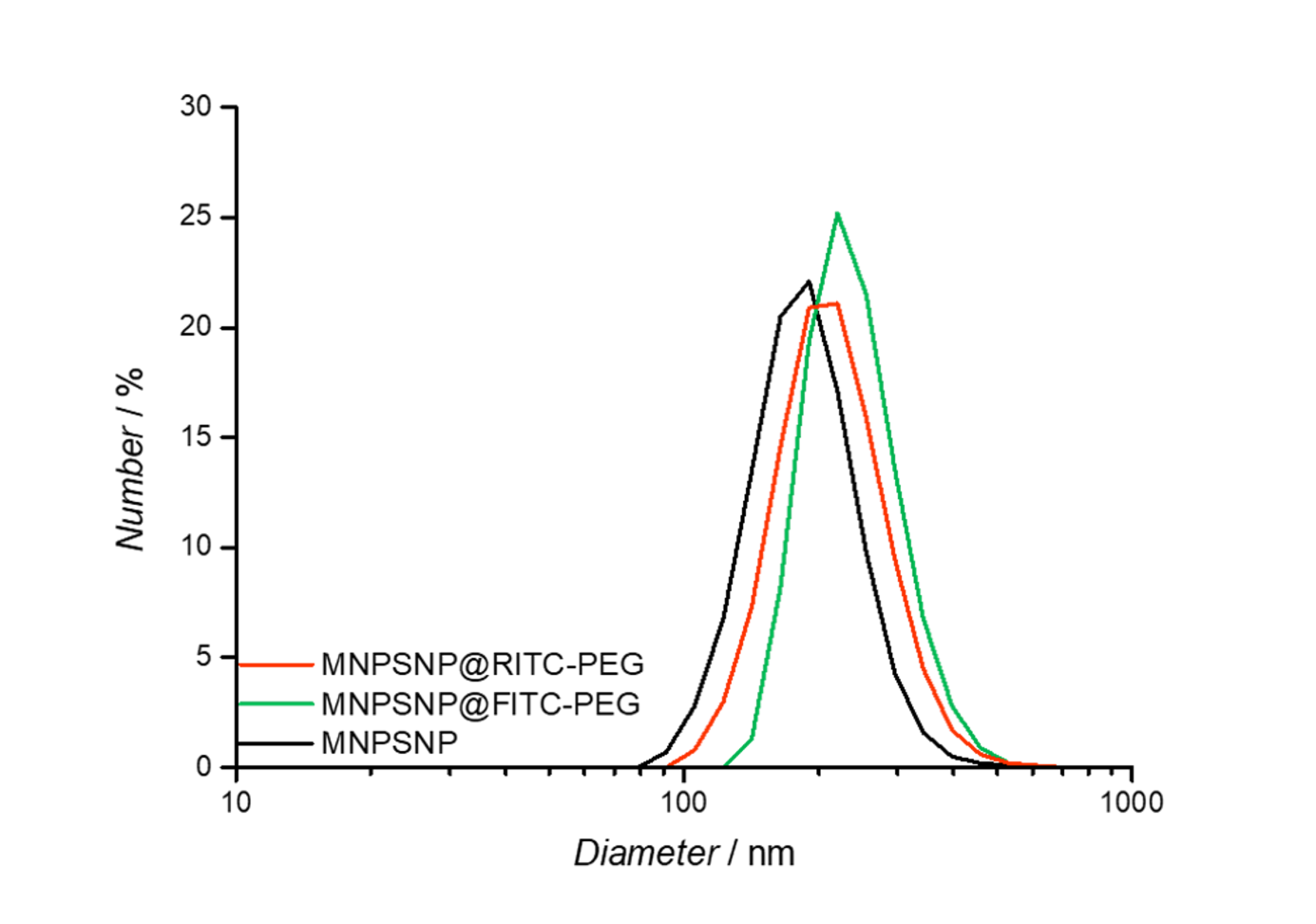


Figure S3: Dynamic light scattering measurements of unmodified MNPSNPs (black), MNPSNP@FITC-PEG (green) and MNPSNP@RITC-PEG (orange) in ultrapure water.

Table S1: Dynamic light scattering measurements and Zeta potential of unmodified MNPSNPs, MNPSNP@FITC-PEG and MNPSNP@RITC-PEG in ultrapure water. PDI is the polydispersity index.

| Sample | Diameter / nm | PDI |  | Zeta potential / mV |
| --- | --- | --- | --- | --- |
| MNPSNP | 191 | 0.346 |  | −33.6 |
| MNPSNP@FITC-PEG | 243 | 0.364 |  | −30.0 |
| MNPSNP@RITC-PEG | 220 | 0.355 |  | −29.6 |
